# Lactylated SPTAN1 Accelerates Hepatocellular Carcinoma Progression by Promoting NOTCH1/HES1 Activation and Immunosuppression

**DOI:** 10.1002/advs.202507068

**Published:** 2025-11-16

**Authors:** Zengbin Wang, Dongjie Ye, Linqing Wu, Jiayu Liu, Banglun Pan, Zhu Zhang, Xiaoxia Zhang, Yuxin Yao, Nanhong Tang

**Affiliations:** ^1^ Department of Immunology School of Basic Medical Sciences Fujian Medical University Fuzhou 350122 China; ^2^ Department of Hepatobiliary Surgery and Fujian Institute of Hepatobiliary Surgery Fujian Medical University Union Hospital Fuzhou 350001 China; ^3^ Department of Physiology and Pathophysiology School of Basic Medical Sciences Fujian Medical University Fujian 350122 China; ^4^ Cancer Center of Fujian Medical University Fujian Medical University Union Hospital Fuzhou 350001 China; ^5^ Key Laboratory of Gastrointestinal Cancer (Fujian Medical University) Ministry of Education Fuzhou 350122 China; ^6^ Key Laboratory of Clinical Laboratory Technology for Precision Medicine (Fujian Medical University) Fujian Province University Fuzhou 350122 China

**Keywords:** CD8^+^ T_ex_, HCC, lactylation, NOTCH1/HES1, SPTAN1

## Abstract

Nonerythrocytic alphaII‐spectrin (SPTAN1) is crucial for neuronal functions, yet its role in oncogenic processes remains inadequately characterized. This study investigates the lactylation (kla) modification of SPTAN1 (SPTAN1‐kla) and its mechanistic contributions to hepatitis B virus (HBV)‐related hepatocellular carcinoma (HCC). Results indicate that SPTAN1 undergoes lactylation at lysine residues K1952 and K1957 specifically in HBV‐positive HCC tissues. Alanyl‐tRNA synthetase 1 (AARS1) mediates SPTAN1‐kla formation, while histone deacetylase 1 (HDAC1) acts as a delactylase. HBV infection enhances lactate production by inducing the expression of HK2, promoting SPTAN1‐kla formation, and disrupting the liquid‐liquid phase separation (LLPS) of cytoplasmic SPTAN1, thereby facilitating its nuclear translocation. Within the nucleus, SPTAN1‐kla interacts with core‐binding factor β (CBFB) to activate NOTCH1/HES1 signaling, thereby promoting HCC cell proliferation. Furthermore, SPTAN1‐kla activates the COX2/mPGES1 pathway through NOTCH1/HES1 signaling, thereby enhancing the biosynthesis of prostaglandin E2 (PGE2) and increasing the infiltration of exhausted CD8⁺ T cells. Therapeutic targeting of SPTAN1‐kla using specific inhibitory peptides significantly attenuates HCC tumor growth in preclinical models. Our research identifies SPTAN1‐kla as a novel oncogenic driver in HBV‐related HCC, functioning via metabolic reprogramming and immune modulation. These findings position SPTAN1‐kla as a promising therapeutic target for developing precision interventions against HBV‐related HCC.

## Graphical abstract

This study identifies SPTAN1‐kla as a novel oncogenic driver in HBV‐driven HCC, linking metabolic, epigenetic, and immune mechanisms. SPTAN1‐kla promotes HCC progression via NOTCH1/HES1 activation and immunosuppression. Findings highlight that targeting SPTAN1‐kla provides a framework for HCC treatment to improve clinical outcomes in HBV infected patients.

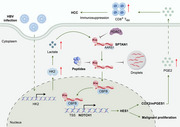

## 1 Introduction

Lactylation (kla) is a significant post‐translational modification (PTM) characterized by the addition of lactate groups to lysine residues in proteins,^[^ ^1^ ^]^ playing a crucial role in regulating tumor cell immune evasion and metabolic reprogramming.^[^ ^2^ ^]^ In the context of the association between histone kla and hepatocellular carcinoma (HCC), it has been documented that lactylation at histone H3 lysine 9 and lysine 18 (H3K9la and H3K18la) facilitates the progression of HCC.^[^ ^3^ ^]^ Furthermore, H3K18la is involved in regulating the transcription of key genes in hepatic stellate cells (HSCs), including α‐SMA, Col1a1, and Timp1.^[^ ^4^ ^]^ Non‐histone kla is also prevalent in HCC, predominantly associated with cellular metabolism and cytoskeletal proteins.^[^ ^5^ ^]^ However, the specific role of lactylation of cytoskeletal proteins in HCC progression remains unclear.

Nonerythrocytic alphaII‐spectrin (SPTAN1), a critical cytoskeletal protein, is essential for maintaining cell polarity and stability, exhibiting a high degree of sequence homology (98.42%) between human and mouse genes.^[^ ^6^ ^]^ SPTAN1 comprises 22 domains, with domains 1–9 and 11–21 characterized as typical three‐helix repeat sequences of spectrin. Domain 11 is characterized by the presence of cleavage sites for calpain and binding sites for calmodulin, whereas domain 22 possesses the capability to bind to actin, thereby playing a role in the maintenance of the cytoskeletal system.^[^ ^7^ ^]^ SPTAN1 demonstrates a distinct effect on subcellular localization within tumor cells.^[^ ^8^ ^]^ SPTAN1 interacts with actin at the cell membrane to uphold cell polarity and contact inhibition, thereby limiting tumor progression. In contrast, SPTAN1 promotes tumor cell proliferation and invasion within the cytoplasm. This dichotomy highlights the diverse functions of SPTAN1 based on its subcellular localization, generating significant interest in its role within the nuclei of tumor cells.

In this study, we elucidate the pivotal role of SPTAN1‐kla in the progression of HBV‐related HCC, providing valuable insights for the development of targeted SPTAN1‐kla therapies in HCC.

## 2 Results

### 2.1 The Close Correlation between HBV and Lactylation

**Figure 1:**
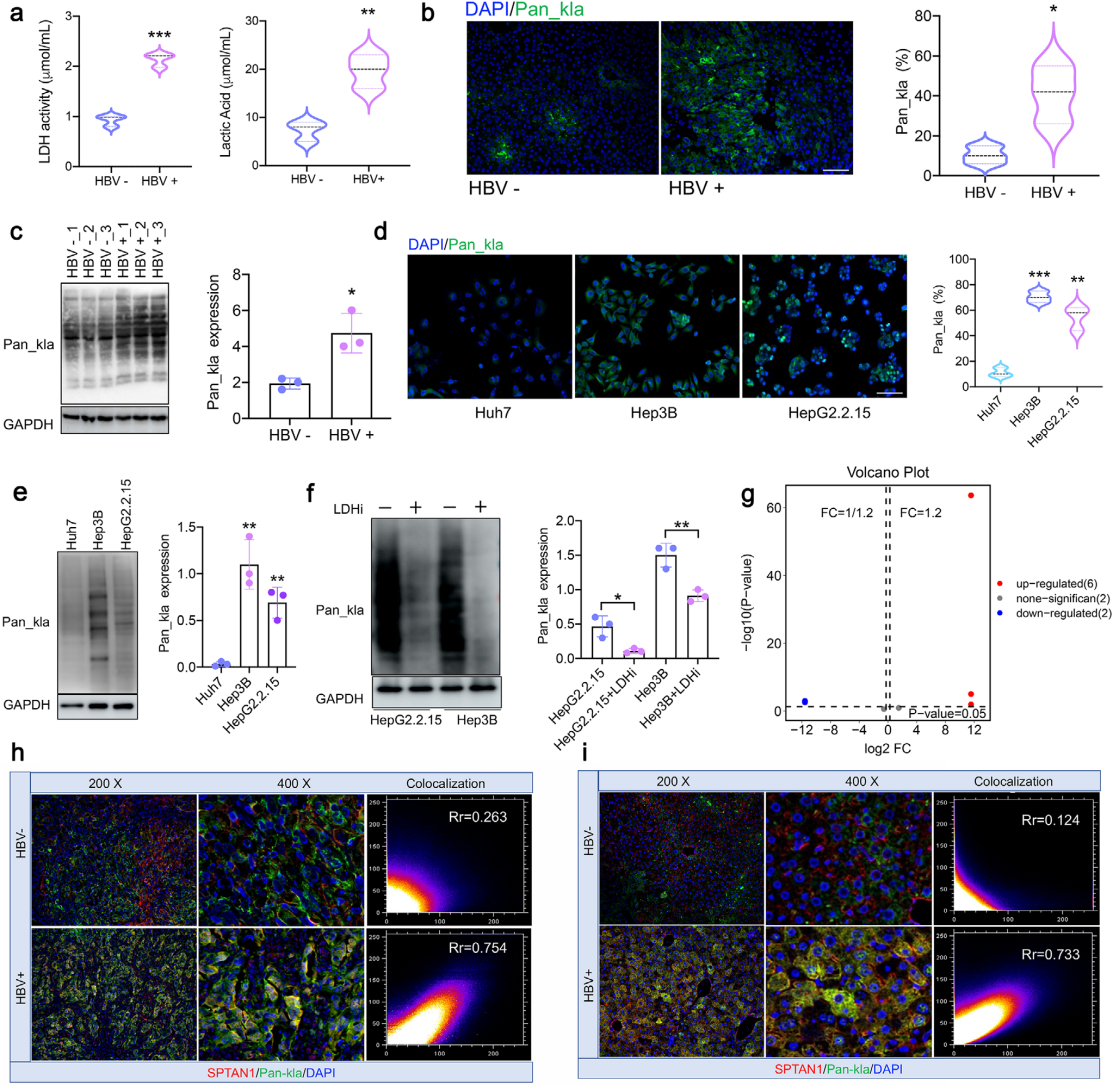
The close correlation between HBV and lactylation. a) LDH activity and lactate concentration in mouse liver cancer tissue. *n* = 3. Data were mean ± SD and analyzed by an unpaired *t*‐test. b,c) Immunofluorescence (IF) and Western Blot detection of Pan‐kla levels in mouse liver cancer tissue. *n* = 3. Data were mean ± SD and analyzed by an unpaired *t*‐test. Bar = 50 µm. d,e) The Pan‐kla levels were measured in HBV^+^HCC cells (Hep3B, HepG2.2.15) and HBV^−^HCC cells (Huh7) using IF d) and Western Blot e), respectively. *n* = 3. Data were mean ± SD and analyzed by ANOVA. Bar = 50 µm. f) Western Blot was used to detect the effect of LDHi on Pan‐kla levels. *n* = 3. Data were mean ± SD and analyzed by an unpaired *t*‐test. g) Label free lactate metabolomics analysis showed volcano plots of kla differential sites. Compared to HBV^−^HCC tissue, HBV^+^HCC tissue upregulated 6 kla sites and downregulated 2 kla sites. *n* = 3. h,i) Co‐localization analysis of SPTAN1 (red) and Pan‐kla (green) in human h) and mouse i) liver cancer tissues. *p < 0.05, **p < 0.01, ***p < 0.001.

We developed the HBV^+^HCC model based on the previously established primary HCC model^[^ ^9^ ^]^ and assessed its efficacy through liver function tests (Table S1, Supporting Information) and the five hepatitis B assays (Table S2, Supporting Information). Our findings indicate that the serum lactate dehydrogenase (LDH) and lactate levels were significantly elevated in the HBV^+^HCC group compared to the HBV^−^HCC group (**Figure** **1**a). Moreover, the Pan‐kla level in the HBV^+^HCC group was significantly higher than that in the HBV^−^HCC group (Figure 1b,c). Additionally, in vitro experiments confirmed that Pan‐kla levels in HBV^+^HCC cells (Hep3B and HepG2.2.15) were significantly higher than in HBV^−^HCC cells (Huh7) (Figure 1d,e). The application of lactate dehydrogenase inhibitors (LDHi) markedly reduced Pan‐kla levels (Figure 1f). These results suggest that HBV facilitates lactylation by inducing lactate production.

Previous studies have shown that HBV stimulates lactate production.^[^ ^10^ ^]^ HBV facilitates the process of kla, but which proteins are involved in this kla process? To investigate this, we conducted a label‐free lactylation modification omics analysis. Compared to HBV^−^HCC tissue, HBV^+^HCC tissue upregulated SPTAN1‐kla, Kif27‐kla, and Ovol2‐kla (Figure 1g). Previous kla omics data showed that SPTAN1‐kla was significantly upregulated in human HBV‐related liver cancer tissues compared to adjacent liver tissues, but Kif27‐kla and Ovol2‐kla were not upregulated.^[^ ^11^ ^]^ Therefore, our research focuses on HBV and SPTAN1‐kla. Further validation was conducted through immunofluorescence (IF) to confirm the co‐localization of SPTAN1 and Pan‐kla in both human and mouse HBV^+^HCC tissues (Figure 1h,i). In addition, co‐immunoprecipitation (Co‐IP) analysis demonstrated that the levels of SPTAN1‐kla in the nucleus of HBV^+^HCC tissues were significantly higher than those in the cytoplasm in both human and mice (Figure S1a,b, Supporting Information). In summary, we hypothesize that HBV‐induced kla is dependent on lactate.

### 2.2 SPTAN1‐kla is Significantly Associated with the Progression of HCC

**Figure 2:**
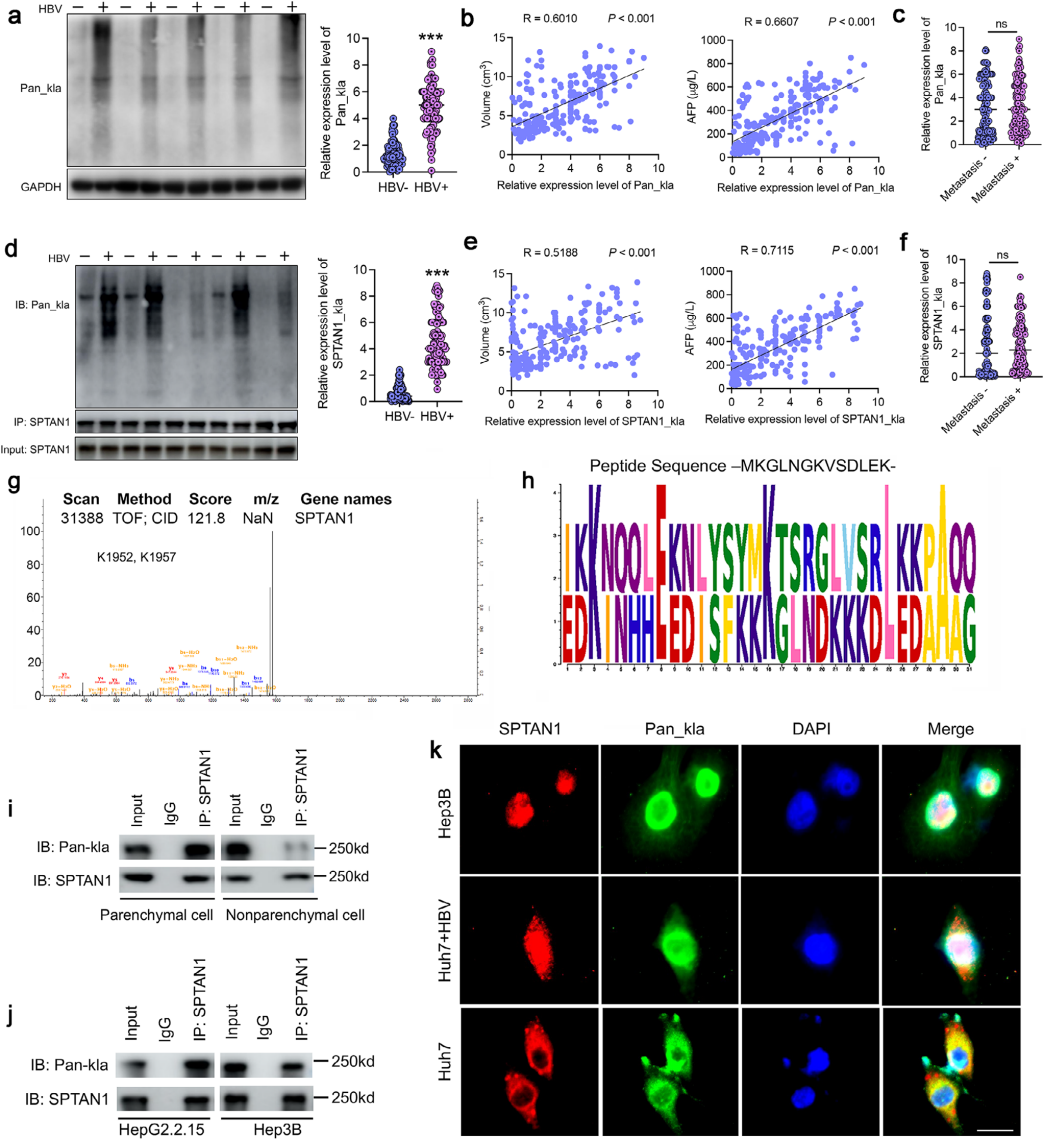
Correlation between SPTAN1‐kla and clinical features of HBV‐related HCC. a) Pan‐kla levels in clinical liver cancer tissues. HBV^−^HCC (*n* = 108), HBV^+^HCC (*n* = 128). Data were mean ± SD and analyzed by an unpaired *t*‐test. b) Pearson correlation analysis of the correlation between Pan‐kla and tumor volume and AFP values. *n* = 236. c) Pan‐kla levels in HCC tissues with and without lymph node metastasis (metastasis+, *n* = 119; metastasis‐, *n* = 117). “ns” indicates no significant difference. d) SPTAN1‐kla levels in clinical liver cancer tissues. HBV^−^HCC (*n* = 108), HBV^+^HCC (*n* = 128). Data were mean ± SD and analyzed by an unpaired *t*‐test. e) Pearson correlation analysis of the correlation between SPTAN1‐kla and tumor volume and AFP values. *n* = 236. f) SPTAN1‐kla levels in HCC tissues with and without lymph node metastasis (metastasis+, *n* = 119; metastasis‐, *n* = 117). “ns” indicates no significant difference. g) Mass spectra of secondary peptide segments at positions K1952 and K1957 of SPTAN1. h) SPTAN1‐kla site motif analysis. i) Co‐IP detection of SPTAN1‐kla (band position 250 kd) in parenchymal and non‐parenchymal cells of HBV^+^HCC liver tissues in mice. *n* = 3. j) Co‐IP detection of SPTAN1‐kla expression in HBV^+^HCC cells (Hep3B and HepG2.2.15). *n* = 3. k) IF analysis of SPTAN1‐kla localization in HBV^+^HCC cells (Hep3B), HBV^−^HCC cells (Huh7) and Huh7 cells infected with HBV. Bar = 5 µm.

To clarify the relationship between Pan‐kla and clinical characteristics of HBV‐related HCC, we collected cancer tissues from 108 HBV^−^HCC and 128 HBV^+^HCC patients. Immunoblot detection demonstrated elevated expression levels of Pan‐kla in HBV^+^HCC tissues (**Figure** **2**a). The Pan‐kla levels in HCC infected with HBV were significantly higher than those in adjacent tissues (Figure S1c, Supporting Information). Pearson correlation analysis showed a positive correlation between Pan‐kla expression and both HCC tumor volume and AFP levels (Figure 2b). However, there was no significant difference in Pan‐kla between HCC tissues with and without lymph node metastasis (Figure 2c). Further clarification of the relationship between SPTAN1‐kla and clinical features of HBV‐related HCC revealed high expression of SPTAN1‐kla in HBV^+^HCC tissues (Figure 2d). SPTAN1‐kla expression was also positively correlated with both HCC tumor volume and AFP levels (Figure 2e). Nonetheless, similar to Pan‐kla, there was no significant difference in SPTAN1‐kla expression between HCC tissues with and without lymph node metastasis (Figure 2f). Therefore, the correlation between these clinical features suggests that SPTAN1‐kla may play a significant role in the progression of HBV‐related HCC.

We further analyzed the modification omics data and identified the kla sites of SPTAN1 as K1952 and K1957 (Figure 2g,h). Notably, the levels of SPTAN1‐kla in the parenchymal cells of HBV^+^HCC tissues were significantly elevated compared to those in non‐parenchymal cells (Figure 2i), suggesting that SPTAN1‐kla may preferentially influence HBV^+^HCC cells rather than immune cells. In addition, SPTAN1‐kla was detected in the HBV^+^HCC cell model (Figure 2j), and HBV infection can promote the localization of SPTAN1‐kla in the nucleus (Figure 2k). Specifically, SPTAN1‐kla was also observed mostly in the nucleus in HBV^+^HCC cells (Hep3B) (Figure 2k). This finding may imply that HBV facilitates the nuclear localization of SPTAN1‐kla.

### 2.3 HBV Induces SPTAN1‐kla Nuclear Translocation

**Figure 3:**
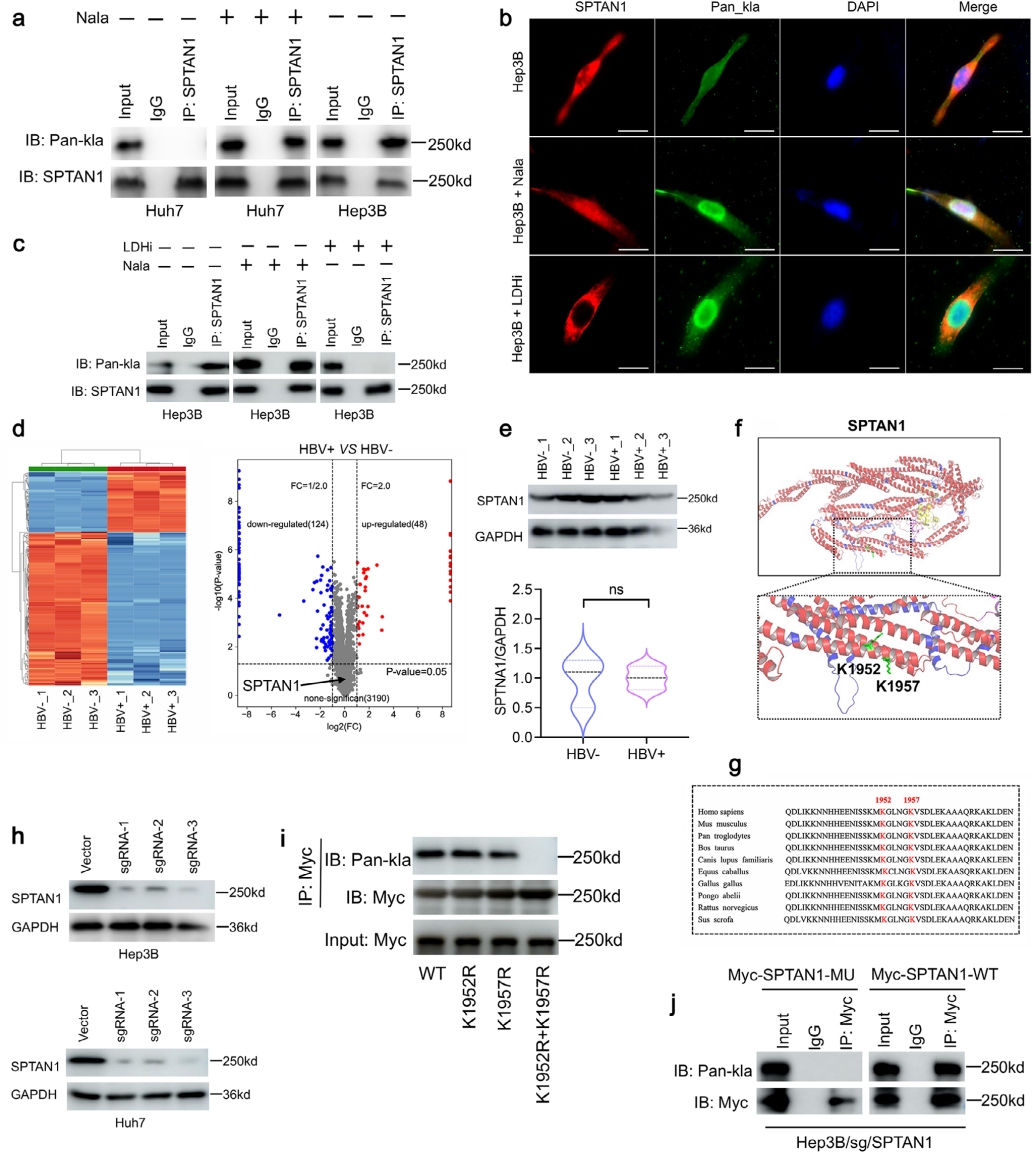
HBV promotes an increase in SPTAN1‐kla in the nucleus. a) HBV^−^HCC cells (Huh7) were treated with Nala (10 mM) for 24 h, and the nuclear SPTAN1‐kla levels were detected by Co‐IP assay. HBV^+^HCC cells (Hep3B) were used as positive controls. *n* = 3. b) The IF experiment was used to evaluate the effects of Nala and LDHi on SPTAN1‐kla. Bar = 5 µm. c) Co‐IP was used to detect SPTAN1‐kla levels in the nucleus. *n* = 3. d) Differential protein analysis of mouse HBV^+^HCC and HBV^−^HCC tissues using label‐free proteomics, displaying clustering heatmaps (left) and differential protein volcano maps (right). e) Western Blot was used to detect the SPTAN1 protein levels. *n* = 3. Data were mean ± SD and analyzed by *t*‐test. f) Conformational analysis of SPTAN1 protein. g) SPTAN1 protein sequence analysis. h) Western Blot validation of SPTAN1 expression levels. *n* = 3. i) Co‐IP detection of SPTAN1‐kla modification sites in Hep3B. *n* = 3. j) Co‐IP validation of modified and mutant plasmids of SPTAN1. *n* = 3. “ns” indicates no significant difference.

To substantiate the hypothesis that HBV induces nuclear translocation of SPTAN1‐kla, we evaluated the effect of lactate promoter Nala on SPTAN1‐kla in HBV^−^HCC cells (Huh7), HBV^+^HCC cells (Hep3B and HepAD38). Our findings indicate that Nala facilitates the expression of SPTAN1‐kla in the nucleus (**Figure** **3**a; Figure S1d, Supporting Information). Notably, the nuclear levels of SPTAN1‐kla in HBV^+^HCC cells were significantly elevated following Nala treatment; however, this effect was mitigated by LDHi (Figure 3b,c). We also found that HBV can promote SPTAN1‐kla in the cell nucleus in a spontaneous mouse model of HBV (Figure S1e, Supporting Information). These findings suggest that HBV enhances nuclear SPTAN1‐kla levels through lactate induction. Nevertheless, the question arises as to whether HBV also influences the expression of the SPTAN1 protein. Our analysis of label‐free proteomics data revealed that HBV does not alter SPTAN1 protein expression (Figure 3d), a finding corroborated by Western blot analysis (Figure 3e). These results propose that HBV modulates the kla level of SPTAN1 rather than its protein expression level.

Since HBV can stimulate lactate production by inducing HK2,^[^ ^12^ ^]^ we next analyzed glycolytic enzymes and lactate transporters by RNA‐seq to clarify the relationship between HBV, lactate, and SPTAN1‐kla. The results showed that HBV promotes the expression of HK1, HK2, LDHA, PKM, TPI1, and SLC16A4 (Figure S2a, Supporting Information). Targeting these genes with siRNA confirmed that HBV increases the level of SPTAN1‐kla by inducing HK2 rather than HK1, LDHA, PKM, TPI1, and SLC16A4 (Figure S2b, Supporting Information). siRNA HK2 significantly reduced HBV‐induced lactate production (Figure S2c, Supporting Information). These findings indicate that HBV triggers the expression of HK2, resulting in lactate generation and, consequently, the induction of SPTAN1‐kla production. Given that SPTAN1 contains two kla sites, it is imperative to ascertain which specific kla site is affected by HBV. Through conformational analysis, we identified that lysines K1952 and K1957 of SPTAN1 are in close proximity and both reside within the 18th domain (Figure 3f). Comparative analysis of SPTAN1 sequences across various species demonstrated strong sequence conservation surrounding the K1952 and K1957 loci (Figure 3g). To eliminate the influence of endogenous SPTAN1, we generated SPTAN1 knockout cell lines (Hep3B/sg/SPTAN1 and Huh7/sg/SPTAN1) using CRISPR/Cas9 technology (Figure 3h). A single point mutation in SPTAN1 did not affect SPTAN1‐kla, whereas a double point mutation resulted in the inhibition of SPTAN1‐kla (Figure 3i). Consequently, Myc‐SPTAN1‐WT was identified as the modified plasmid (SPTAN1‐kla), and Myc‐SPTAN1‐(K1952R + K1957R) was identified as the mutant plasmid (SPTAN1‐MU). SPTAN1‐MU was not modified by kla (Figure 3j). The above results confirm that the kla sites of SPTAN1 are K1952, and K1957, and that HBV promotes an increase in SPTAN1‐kla levels in the nucleus.

### 2.4 Kla Facilitates the Nuclear Import of SPTAN1 by Interfering with Liquid–Liquid Phase Separation

**Figure 4:**
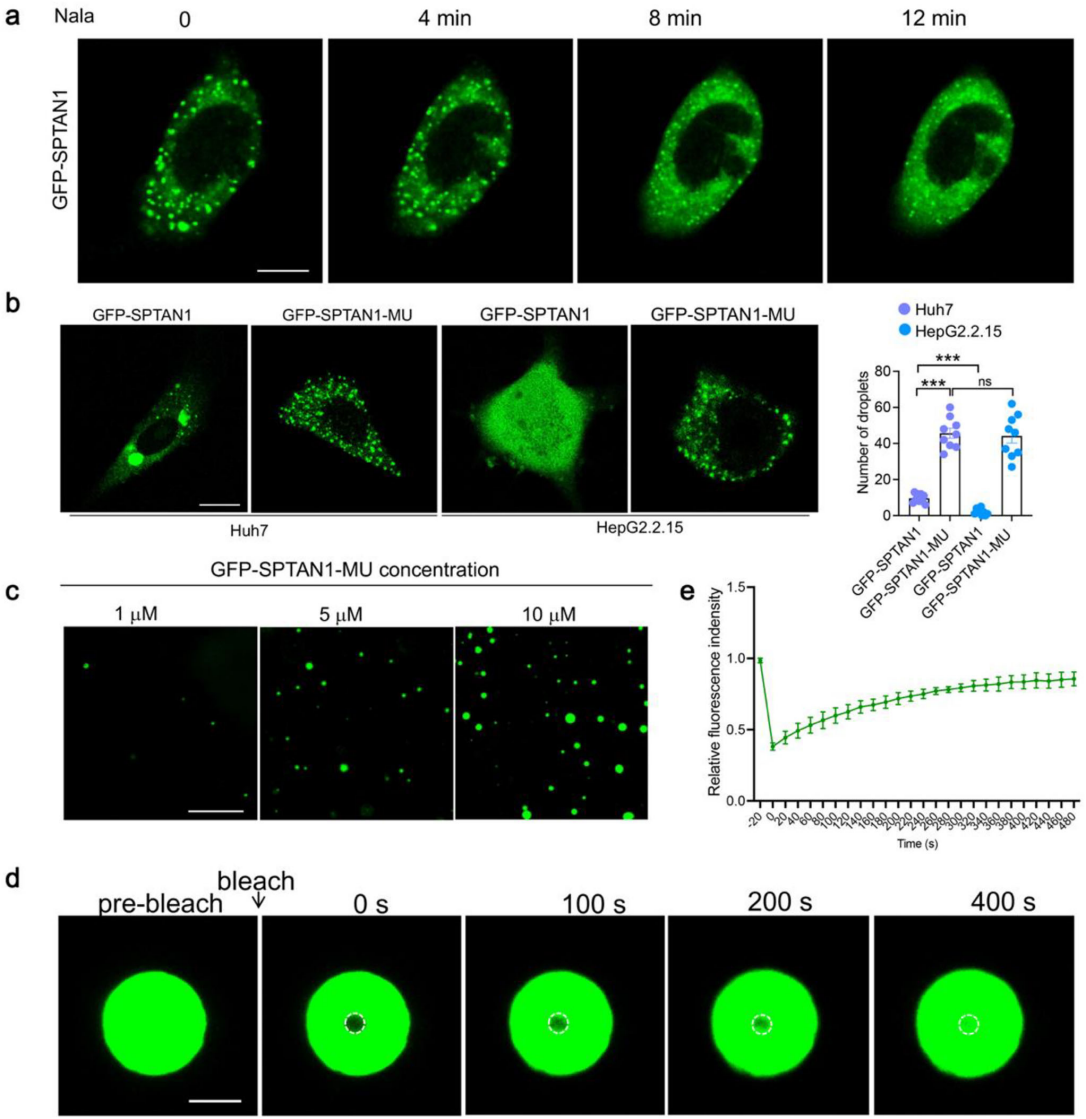
Kla promotes the nuclear import of SPTAN1 by interfering with LLPS. a) Time‐lapse imaging of Huh7 cells expressing GFP‐SPTAN1 treated with Nala for 12 min. Bar = 5 µm. b) The representative living cell images of GFP‐SPTAN1 or GFP‐SPTAN1‐MU expressed in Huh7 and HepG2.2.15 cells. Quantification of SPTAN1 droplets number. *n* = 9. Data were mean ± SD and analyzed by one‐way ANOVA. Bar = 5 µm. c) Cell‐free in vitro phase separation assay showing droplet formation of GFP‐SPTAN1‐MU in different concentrations. *n* = 3. Bar = 10 µm. d,e) FRAP analysis of GFP‐SPTAN1‐MU protein. Quantification of fluorescent intensity of the area indicated by the dashed circle after FRAP experiment. *n* = 9. Bar = 1 µm. “ns” indicates no significant difference. ***p < 0.001.

Our forthcoming investigation will focus on elucidating the mechanisms by which HBV facilitates the elevation of SPTAN1‐kla levels within the nucleus. Previous studies have reported that liquid–liquid phase separation (LLPS) can facilitate the retention of proteins in the cytoplasm, thereby indirectly inhibiting their nuclear import.^[^ ^13^ ^]^ To ascertain whether intranuclear SPTAN1‐kla is associated with LLPS, we constructed expression plasmids for GFP‐SPTAN1 and GFP‐SPTAN1‐MU. Following Nala stimulation, we observed a progressive reduction in SPTAN1 droplets in the cytoplasm, and an increase in droplet‐free intranuclear SPTAN1. This observation suggests that lactate inhibits the LLPS of SPTAN1, thereby preventing the formation of SPTAN1 droplets in the cytoplasm and facilitating its nuclear import (**Figure** **4**a). Subsequently, we transfected Huh7 and HepG2.2.15 cells with GFP‐SPTAN1 and GFP‐SPTAN1‐MU, respectively. Our results demonstrated that the cytosolic droplets of GFP‐SPTAN1‐MU were significantly more abundant than those of GFP‐SPTAN1, indicating a reduction in the formation of droplets induced by HBV‐mediated kla (Figure 4b). Furthermore, we purified the GFP‐SPTAN1‐MU recombinant protein and added it to phase separation buffer at different final concentrations. It was observed that GFP‐SPTAN1‐MU underwent in vitro phase separation, with a rapid increase in the number of droplets as the concentration rose (Figure 4c). GFP‐SPTAN1‐MU demonstrated the ability to undergo fluorescence bleaching recovery, indicating its droplet‐like properties and fluidity (Figure 4d,e). The findings suggest that HBV enhances the accumulation of intranuclear SPTAN1‐kla by inducing kla to interfere with the formation of SPTAN1 droplets.

### 2.5 Activation of NOTCH1/HES1 Signaling by Nuclear SPTAN1‐kla

**Figure 5:**
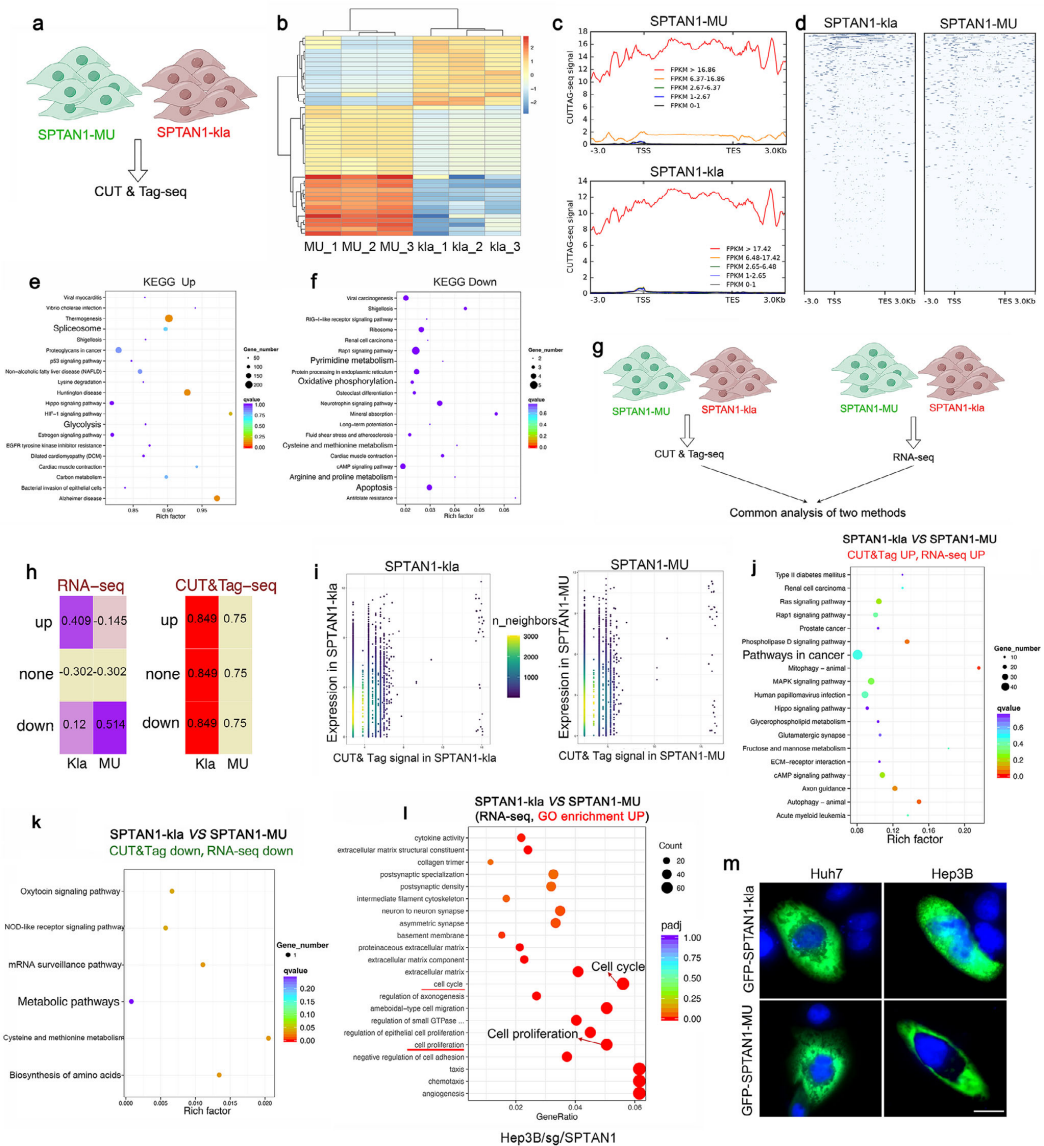
SPTAN1‐kla in the nucleus promotes oncogene expression by binding to their promoters. a) The Hep3B/sg/SPTAN1 cells were transfected with SPTAN1‐kla and SPTAN1‐MU, respectively. CUT&Tag analysis was performed. b) Cluster diagram of enriched peaks in SPTAN1‐kla and SPTAN1‐MU. *n* = 3. c,d) The signal distribution map c) and peak heat map d) of the combination of SPTAN1‐kla and SPTAN1‐MU. e,f) The upregulation and downregulation of peak signals were mainly enriched in Hep3B/sg/SPTAN1 cells transfected with SPTAN1‐kla compared to transfection with SPTAN1‐MU. g) Schematic diagram of CUT&Tag combined with RNA‐seq analysis. *n* = 3. h) CUT&Tag combined with RNA‐seq analysis showed an overview of the effects of SPTAN1‐kla and SPTAN1‐MU on gene expression. i) Correlation density scatter plot between CUT&Tag signal and gene expression levels in the promoter subregion. j,k) KEGG was used to analyze the enriched signaling pathways of upregulated and downregulated genes. l) RNA‐seq was used to detect differentially expressed genes in Hep3B/sg/SPTAN1 cells transfected with SPTAN1‐kla and SPTAN1‐MU, and GO enrichment analysis was performed. m) IF was used to detect the localization of SPTAN1‐kla in Hep3B and Huh7 cells. Bar = 5 µm.

Considering that HBV promotes the nuclear entry of SPTAN1‐kla, we speculated that SPTAN1‐kla might function as a transcription factor (TF). To evaluate how nuclear SPTAN1‐kla regulates gene expression, we transfected SPTAN1‐kla and SPTAN1‐MU separately into Hep3B/sg/SPTAN1 cells. Subsequently, we employed CUT&Tag analysis to identify the target genes of SPTAN1‐kla (**Figure** **5**a,b). SPTAN1‐kla exhibited significantly enhanced binding to gene promoter regions compared to SPTAN1‐MU (Figure 5c,d). The upregulated peaks induced by SPTAN1‐kla were predominantly associated with glycolysis and spliceosome signaling (Figure 5e), whereas the downregulated peaks were primarily linked to metabolic and apoptotic signaling pathway (Figure 5f). To further assess the influence of SPTAN1‐kla on gene expression, we performed a combined CUT&Tag and RNA‐seq analysis (Figure 5g). The findings demonstrated that SPTAN1‐kla primarily upregulated gene expression in both RNA‐seq and CUT&Tag analyses (Figure 5h). Moreover, the CUT&Tag signal at the promoter regions exhibited a stronger correlation with gene expression levels for SPTAN1‐kla, suggesting that SPTAN1‐kla upregulates target gene expression by binding to promoter regions (Figure 5i). KEGG enrichment analysis revealed that the genes upregulated by SPTAN1‐kla were mainly associated with cancer pathways (Figure 5j), while the downregulated genes were chiefly related to metabolic pathways (Figure 5k). Additionally, SPTAN1‐kla increased cell proliferation and enrichment of cell cycle signaling pathways in HBV‐positive cells (Figure 5l), but did not have this effect in HBV‐negative cells (Figure S3a, Supporting Information). The CCK‐8 experiment confirmed that SPTAN1‐kla promotes the proliferation of HBV‐positive cells without affecting the proliferation of HBV‐negative cells (Figure S3b, Supporting Information). SPTAN1‐kla was found to localize within the nucleus of Hep3B cells (Figure 5m). Thus, SPTAN1‐kla in the nucleus may activate and promote the expression of oncogenes by binding to their promoters.

To elucidate the genes affected by SPTAN1‐kla, further research revealed that SPTAN1‐kla enhances signaling at the promoter binding regions of NOTCH1 and HES1 (Figure S4a, Supporting Information), thereby augmenting the expression of these genes. In contrast, SPTAN1‐kla attenuates signaling at the promoter binding region of adenosyltransferase 2A (MAT2A) gene (Figure S4a, Supporting Information), resulting in reduced MAT2A expression. MAT2A serves as a critical enzyme in the conversion of methionine (Met) to S‐adenosylmethionine (SAM), a substrate essential for DNA methylation,^[^ ^14^ ^]^ suggesting a potential interplay between SPTAN1‐kla and MAT2A‐mediated DNA methylation processes. Furthermore, RNA‐seq data also demonstrated that SPTAN1‐kla upregulates NOTCH1 and HES1 expression while concurrently downregulating MAT2A expression (Figure S4b, Supporting Information). Additionally, previous studies have established a correlation between NOTCH1/HES1 and the expression of proliferation‐related proteins, such as CDK4 and Ki67.^[^ ^15^ ^]^

In light of these considerations, we proposed the hypothesis that SPTAN1‐kla might regulate DNA methylation through MAT2A, thereby modulating the expression of NOTCH1 and HES1, and subsequently activating the NOTCH1/HES1 signaling pathway, which could lead to an upregulation of genes associated with cell proliferation. To validate this hypothesis, we performed methylation sequencing. Contrary to our expectations, cells transfected with either SPTAN1‐kla or SPTAN1‐MU exhibited nine CpG sites within the CpG island of the HES1 promoter, with no significant difference in methylation levels at each site (Figure S4c; Table S3, Supporting Information). Furthermore, we found no significant difference in methylation levels of the NOTCH1 promoter (Figure S4d, Supporting Information). These findings suggest that SPTAN1‐kla does not affect the promoter methylation of NOTCH1 and HES1.

### 2.6 SPTAN1‐kla Activates NOTCH1/HES1 Signaling Through Interaction with CBFB

**Figure 6:**
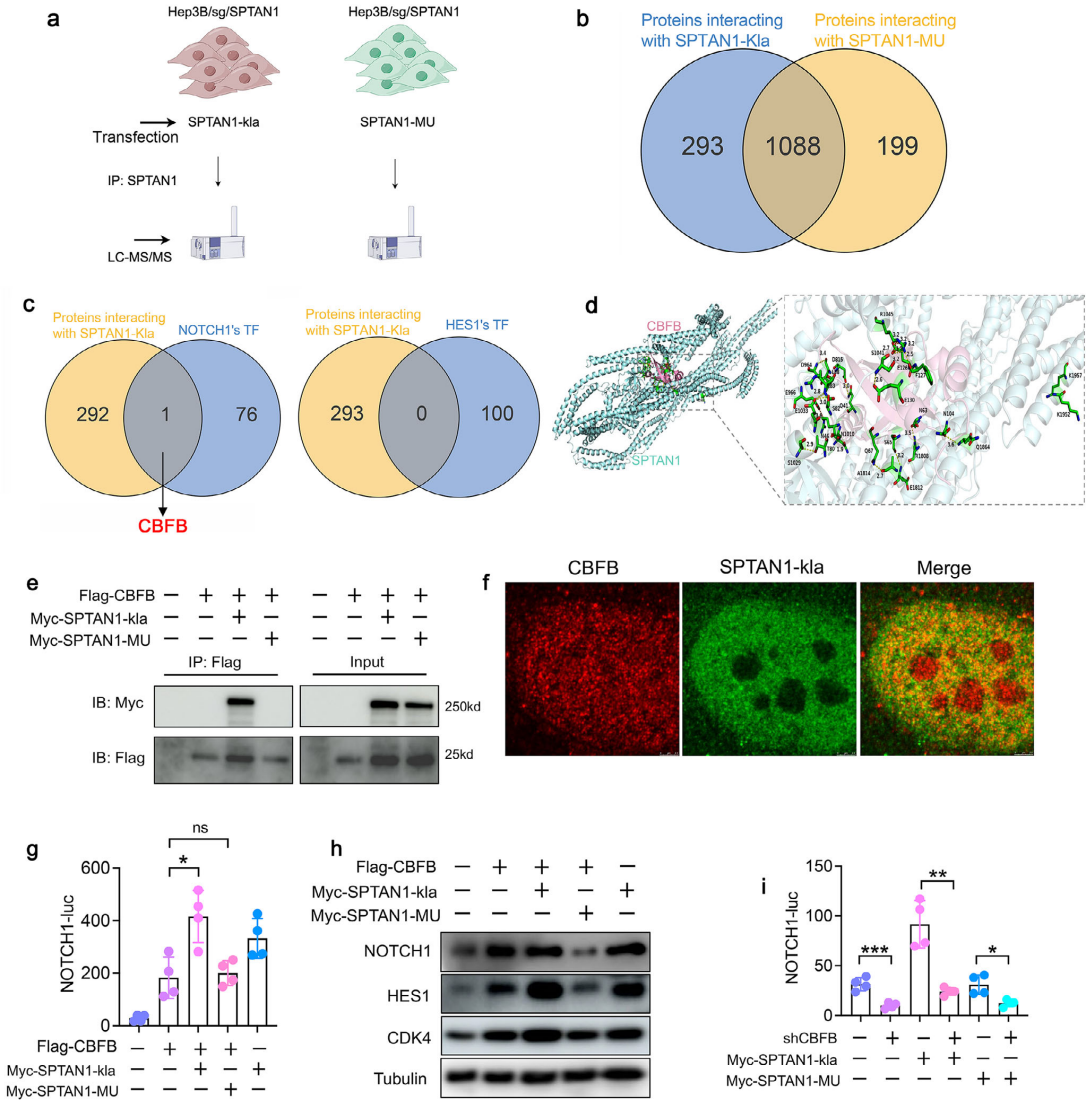
SPTAN1‐kla activates NOTCH1/HES1 signaling via interaction with CBFB. a) Schematic diagram of SPTAN1‐kla and SPTAN1‐MU transfection into Hep3B/sg/SPTAN1 cells for LC‐MS/MS analysis. b) Venn diagram showing proteins interacting with SPTAN1‐kla and SPTAN1‐MU, respectively. c) Comparison of 77 TFs of NOTCH1 and 100 TFs of HES1 screened from the hTFtarget database with the 293 proteins interacting uniquely with SPTAN1‐kla. d) Molecular docking revealed multiple hydrogen bonding interactions between SPTAN1‐kla and CBFB. The yellow dashed line represents hydrogen bonds. e) Co‐IP confirming the interaction between CBFB and SPTAN1‐kla. *n* = 3. f) IF assay showing colocalization of CBFB with SPTAN1‐kla within the nucleus. *n* = 4. g) DLR assay detecting NOTCH1 promoter activity. *n* = 4. Data were mean ± SD and analyzed by ANOVA. h) Western blot analysis detecting protein expression of NOTCH1, HES1, and CDK4. *n* = 3. i) DLR assay detecting NOTCH1 promoter activity. *n* = 4. Data were mean ± SD and analyzed by *t*‐test. *p < 0.05, **p < 0.01, ***p < 0.001.

Given that SPTAN1‐kla does not alter the methylation modification status of the NOTCH1 and HES1 promoters, we explored the possibility that nuclear‐localized SPTAN1‐kla might interact with TF associated with NOTCH1 and HES1. To investigate this, we conducted an LC‐MS/MS analysis (**Figure** **6**a). We found that SPTAN1‐kla interacted with 1381 proteins, whereas SPTAN1‐MU interacted with 1287 proteins. A comparative analysis identified 293 proteins that uniquely interacted with SPTAN1‐kla (Figure 6b). Using the hTFtarget database, we screened for TF associated with NOTCH1 and HES1, identifying 77 TF for NOTCH1 and 100 TF for HES1. Notably, core binding factor beta (CBFB) emerged as a common TF intersection for SPTAN1‐kla and NOTCH1, but not for HES1 (Figure 6c). Additionally, RNA‐seq data indicated that SPTAN1‐kla did not affect CBFB expression (Figure S4b, Supporting Information). Previous works have demonstrated that CBFB can potentiate the oncogenic activity of NOTCH1.^[^ ^16^ ^]^ Consequently, we speculated that SPTAN1‐kla promotes the activation of NOTCH1/HES1 signaling by interacting with CBFB. Molecular docking has revealed multiple hydrogen bonding interactions between SPTAN1‐kla and CBFB (Figure 6d). Co‐IP further confirmed the significant interaction between SPTAN1‐kla and CBFB, whereas no significant interaction was observed between SPTAN1‐MU and CBFB (Figure 6e). The single site of SPTAN1 mutation (K1952 or K1957) did not interact with CBFB (Figure S5a, Supporting Information). IF analysis additionally demonstrated the colocalization of GFP‐SPTAN1‐kla and CBFB within the nucleus (Figure 6f). To clarify how SPTAN1‐kla mediates NOTCH1 expression, we conducted ChIP‐seq experiments. It was found that in SPTAN1 knockout cells transfected with SPTAN1‐kla, CBFB could bind to the promoter of the NOTCH1 gene, while in SPTAN1 knockout cells transfected with SPTAN1‐MU, CBFB did not have this effect (Figure S5b,c, Supporting Information). Co‐transfection of CBFB with GFP‐SPTAN1‐kla resulted in enhanced promoter activity of NOTCH1 and increased expression of NOTCH1, HES1 and CDK4 (Figure 6g,h). Moreover, knockdown of CBFB interfered with the NOTCH1 signaling activated by SPTAN1‐kla (Figure 6i). NOTCH1 inhibitors (Tangeretin) significantly inhibited the cancer‐promoting effect of SPTAN1‐kla (Figure S6a, Supporting Information). Genetic ablation of NOTCH1 significantly attenuates SPTAN1‐kla‐mediated enhancement of HCC cell proliferation in vitro (Figure S6b, Supporting Information). Collectively, these results indicate that SPTAN1‐kla promotes the progression of HCC by activating the NOTCH1/HES1 signaling pathway through interaction with CBFB.

To elucidate the enzymes implicated in SPTAN1‐kla, we analyzed the above LC‐MS/MS data, which revealed an enrichment of alanyl‐tRNA synthetase 1 (AARS1), histone deacetylase 1 (HDAC1) and HDAC2. AARS1 was significantly more enriched in the SPTAN1‐kla group than in the SPTAN1‐MU group. Co‐IP results demonstrated interactions between SPTAN1 and both AARS1 and HDAC1, whereas HDAC2 did not show such an interaction (Figure S7a, Supporting Information). Subsequent analysis of shRNA targeting AARS1 and HDAC1 revealed that AARS1 shRNA markedly inhibited SPTAN1‐kla (Figure S7b, Supporting Information), whereas HDAC1 shRNA significantly enhanced SPTAN1‐kla (Figure S7c, Supporting Information). We further investigated the effects of shRNAs targeting AARS1 and HDAC1 on SPTAN1 acetylation and ubiquitination. The results showed that AARS1 shRNA did not affect SPTAN1 acetylation and ubiquitination (Figure S7d, Supporting Information), while HDAC1 shRNA inhibited SPTAN1 acetylation but did not affect SPTAN1 ubiquitination (Figure S7e, Supporting Information). Furthermore, treatment with aminoacyl tRNA synthetase‐IN‐1 was found to significantly suppress the expression of NOTCH1, HES1, and CDK4 (Figure S7f, Supporting Information). In summary, our findings have identified the enzymes responsible for the modification and demodification of SPTAN1‐kla.

### 2.7 SPTAN1‐kla Enhances PGE2 Secretion to Promote HCC Immunosuppression by Inducing CD8^+^ Tex Cell Infiltration

**Figure 7:**
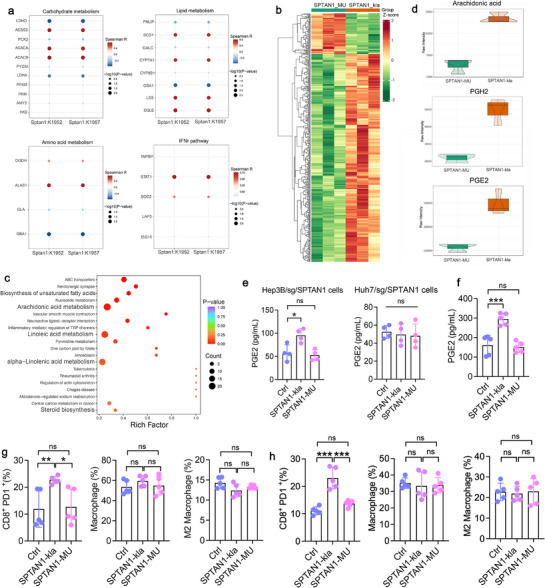
SPTAN1‐kla potentiates PGE2 secretion, thereby fostering immunosuppression in HCC. a) Correlation analysis between the modification levels of SPTAN1's K1952 and K1957 sites and metabolic signals. b) Metabolite clustering heatmap. *n* = 3. c) KEGG enrichment analysis. d) Comparison of arachidonic acid, PGE2, and PGH2 abundance between SPTAN1‐kla and SPTAN1‐MU groups. e) ELISA detection of PGE2 secretion levels in cell supernatants. *n* = 4. Data were mean ± SD and analyzed by ANOVA. f) ELISA detection of PGE2 secretion levels in mouse serum. *n* = 5. Data were mean ± SD and analyzed by ANOVA. g,h) FCM analysis of the ratio of CD8^+^ PD1^+^ cells, Macrophages, and M2 macrophages in mouse subcutaneous HCC established based on Hepa1‐6/sg/SPTAN1 cells g) and H22/sg/SPTAN1 cells h). *n* = 5. Data were mean ± SD and analyzed by ANOVA. “ns” indicates no significant difference. *p < 0.05, **p < 0.01, ***p < 0.001.

The KEGG enrichment results have indicated that SPTAN1‐kla influences cellular metabolism. Correlation analysis further showed a positive correlation between SPTAN1‐kla levels and the expression of several key enzymes involved in carbohydrate and lipid metabolism, including ACSS2, ACACA, ACACB, SCD1, CYP7A1, LSS, and SQLE (**Figure** **7**a). This finding implies that SPTAN1‐kla may influence cellular glucolipid metabolism. Consequently, we conducted untargeted metabolomics sequencing to explore the metabolites affected by SPTAN1‐kla (Figure 7b). The analysis revealed that Hep3B/sg/SPTAN1 cells transfected with SPTAN1‐kla exhibited enriched signals associated with fatty acid biosynthesis, arachidonic acid metabolism, and linoleic acid metabolism (Figure 7c). Notably, SPTAN1‐kla promotes the production of PGE2 and its synthetic precursors arachidonic acid and PGH2 (Figure 7d). ELISA assays confirmed that the level of PGE2 in the supernatants of Hep3B/sg/SPTAN1 cells transfected with SPTAN1‐kla was significantly elevated compared to that in cells transfected with SPTAN1‐MU (Figure 7e). However, this phenomenon was not observed in Huh7/sg/SPTAN1 cells (Figure 7e).

Since SPTAN1‐kla promotes PGE2 secretion, and considering that PGE2 produced by tumor cells can restrict the proliferation and effector functions of CD8^+^ T cells, thereby facilitating cancer immune evasion,^[^ ^17^ ^]^ our previous studies have also shown the relationship between PGE2 and immune suppression in HBV‐related HCC.^[^ ^18^ ^]^ Moreover, inhibiting PGE2 signaling in tumors has been shown to improve the efficacy of PD‐1 blockade.^[^ ^19^ ^]^ We hypothesized that HBV‐driven SPTAN1‐kla might induce functional inhibition of CD8^+^ T cells by promoting PGE2 metabolism and secretion. To validate this hypothesis, we utilized Hepa1‐6 and H22 cells infected with HBV to establish Hepa1‐6/sg/SPTAN1 and H22/sg/SPTAN1 cells, which were subsequently transfected with either SPTAN1‐kla or SPTAN1‐MU, respectively. In the subcutaneous tumor model, ELISA analysis revealed a statistically significant elevation of serum PGE2 concentrations following SPTAN1‐kla treatment (Figure 7f). FCM analysis found that SPTAN1‐kla promoted an increase in exhausted CD8^+^ T (CD8^+^ T_ex_) cells but did not affect macrophages or M2 macrophages (Figure 7g,h). In addition, we analyzed the expression correlation between SPTAN1‐kla, NOTCH1, HES1, and CD8 in human HCC tissues. The findings demonstrated a positive correlation between SPTAN1‐kla expression and both NOTCH1 and HES1, while a negative correlation was observed with CD8 (Figure S8, Supporting Information). These results indicate that SPTAN1‐kla enhances PGE2 secretion levels in HCC cells, induces an increase in CD8^+^ T_ex_ cells, and promotes immunosuppression.

To further clarify the regulatory relationship between NOTCH1/HES1 activated by SPTAN1‐kla and PGE2, we used multiple inhibitors through in vivo and in vitro experiments. Western blot showed that SPTAN1‐kla promoted the expression of COX2 and mPGES1 (Figure S9a, Supporting Information), while the use of siNOTCH1 or siHES1 inhibited this promoting effect (Figure S9a, Supporting Information) and also suppressed the secretion of PGE2 induced by SPTAN1‐kla (Figure S9b, Supporting Information). Nonetheless, SPTAN1‐kla failed to enhance PGE2 secretion in NOTCH1 knockout cells (Figure S9c, Supporting Information), indicating that the NOTCH1/HES1 signaling pathway, which is activated by SPTAN1‐kla, facilitates PGE2 secretion through the induction of COX2/mPGES1. In particular, exogenous addition of PGE2 did not activate the expression of NOTCH1, HES1 and CDK4 (Figure S9d, Supporting Information). Furthermore, the combined use of the NOTCH1 inhibitor (OMP 52M51) and COX2 inhibitor (SC‐58125) reduced the proliferation of HCC cells more effectively than their use alone in cells with overexpression of SPTAN1‐kla (Figure S9e, Supporting Information), indicating that the NOTCH1 and PGE2 pathways synergistically promote HCC. Moreover, in vivo experiments have also confirmed that the combined use of NOTCH1 inhibitors and COX2 inhibitors significantly decreased the infiltration of CD8⁺ T_ex_ cells compared with their use alone (Figure S9f, Supporting Information). These results indicate that SPTAN1‐kla promotes HCC proliferation and immune escape through the synergistic effect of the NOTCH1/HES1 and PGE2 pathways.

### 2.8 Targeting SPTAN1‐kla Inhibits the Progression of HBV‐Related HCC

**Figure 8:**
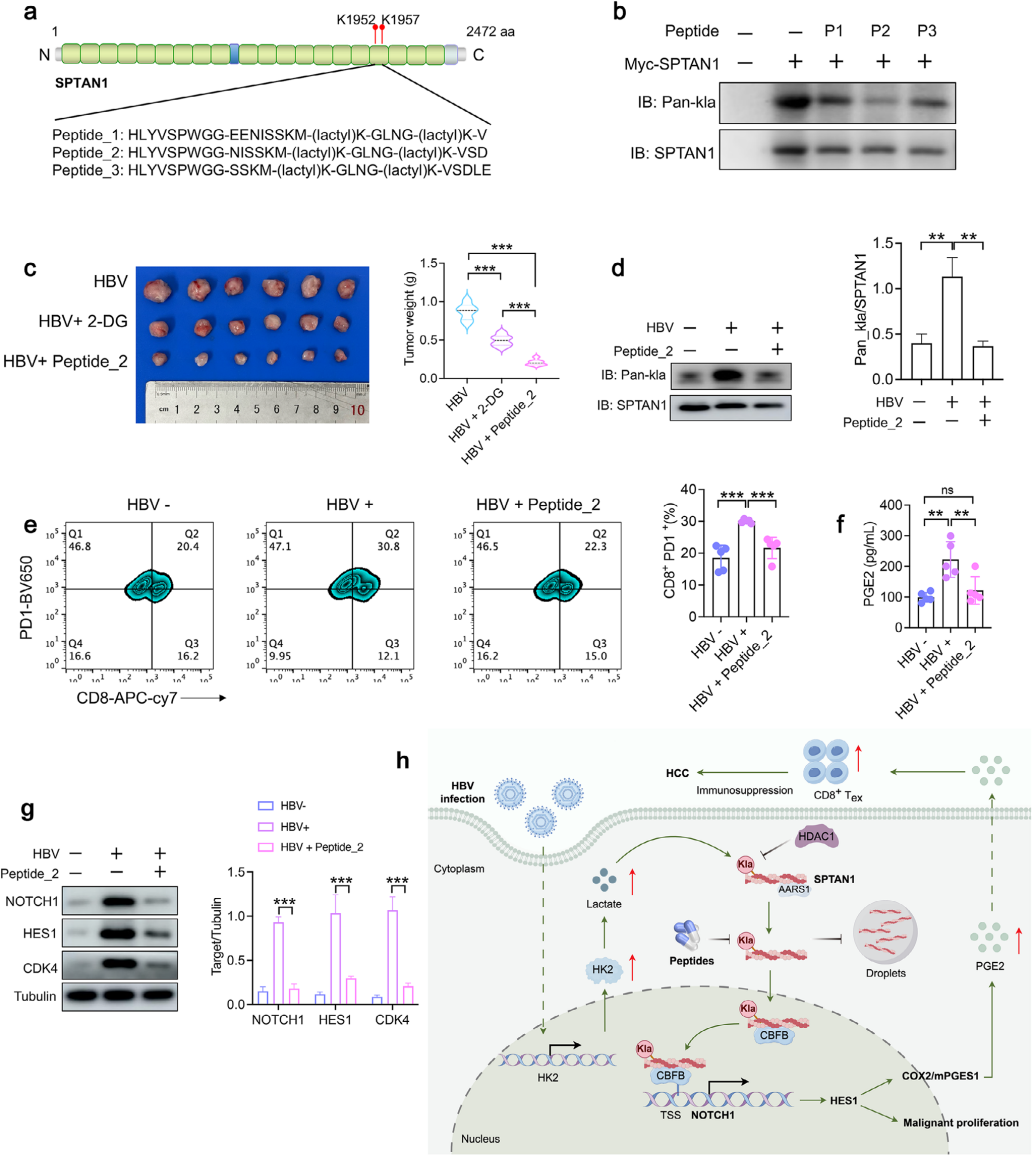
Targeting SPTAN1‐kla inhibits the progression of HBV‐related HCC. a) Schematic diagram of the design of three peptides specifically targeting two modification sites of SPTAN1. b) Co‐IP analysis to identify the peptide with the optimal inhibitory effect on SPTAN1‐kla. c) Hepa1‐6 cells infected with HBV were used to establish a subcutaneous HCC model, and the mice were treated with 2‐DG and peptide_2, respectively. *n* = 6. Data were analyzed by Tukey's HSD test. d) HCC cells were obtained from subcutaneous tumors for further Co‐IP analysis to assess the inhibitory effect of peptide_2 treatment on SPTAN1‐kla. *n* = 3. Data were analyzed by the Dunnet*t*‐test. e) FCM analysis of changes in the proportion of CD8^+^ PD1^+^ cells. *n* = 5. Data were analyzed by the Dunnet*t*‐test. f) ELISA analysis of PGE2 secretion levels in mouse serum. *n* = 5. Data were analyzed by Tukey's HSD test. g) Western blotting to detect the expression levels of NOTCH1, HES1, and CDK4 proteins. *n* = 3. Data were mean ± SD and analyzed by *t*‐test. “ns” indicates no significant difference. *p < 0.05, **p < 0.01, ***p < 0.001. h) The pattern diagram illustrates the mechanism by which SPTAN1‐kla promotes HBV‐related HCC. This image was drawn by Figdraw.

Cell‐penetrating peptides (CPPs) can achieve the effect of inhibiting the target protein kla by competing with kla for binding.^[^ ^20^ ^]^ As illustrated in Figure 8a, we synthesized three peptides specifically targeting two modification sites of SPTAN1. We evaluated the cytotoxicity of these three peptides in vitro. Cells were treated with peptides of different concentrations (0–100 µm) for 24 h. The results showed that the cell survival rates were all above 85% (Figure S10a, Supporting Information), indicating that the peptides were non‐toxic to the cells. Co‐IP was performed to detect SPTAN1‐kla levels and identify peptide_2 as having the most effective inhibitory impact (Figure 8b). Moreover, peptide_2 has no effect on the lactation modification of multiple histones (H3K18la, H3K9la, H3K14la, H4K8la, H4K12la, and H4K5la) and p53 (Figure S10b, Supporting Information). To elucidate the impact of peptide_2 on the growth of HCC, we evaluated the in vivo safety of peptide_2. We found that peptide_2 did not cause damage to the liver, kidneys or spleen of mice within the concentration range of 0–20 mg kg^−1^ (Figure S10c,d, Supporting Information). Next, we utilized Hepa1‐6 cells infected with HBV to establish a subcutaneous HCC model. The administration of peptide_2 or 2‐DG significantly inhibited HCC growth, with peptide_2 demonstrating a more substantial inhibitory effect (Figure 8c). TUNEL staining further indicated that peptide_2 markedly enhanced apoptosis (Figure S11, Supporting Information). Furthermore, treatment with peptide_2 alone significantly reduced SPTAN1‐kla levels (Figure 8d). Importantly, the HBV^+^HCC group exhibited a significantly higher number of CD8^+^PD1^+^ cells compared to the HBV^−^HCC group, an effect that was mitigated by peptide_2 treatment (Figure 8e). ELISA analysis revealed that peptide_2 treatment significantly reduced HBV‐induced PGE2 production (Figure 8f). Western blotting demonstrated that peptide_2 treatment significantly inhibited the expression of NOTCH1, HES1, and CDK4 proteins in Hepa1‐6 cells infected with HBV (Figure 8g). We also employed orthotopic models and observed that the administration of peptide_2 markedly inhibited tumor growth (Figure S12a, Supporting Information). Notably, the number of CD8^+^PD1^+^ cells was also considerably decreased following peptide_2 treatment (Figure S12b,c, Supporting Information). Additionally, peptide_2 treatment significantly reduced HBV‐induced PGE2 production (Figure S12d, Supporting Information). These results suggest that peptide_2 exerts a significant inhibitory effect on HCC by targeting SPTAN1‐kla.

## 3 Discussion

The complex role of SPTAN1 in neuronal biology is well‐established^[^ ^6^ ^]^; however, its involvement in tumorigenesis remains a subject of considerable interest. This study explored the novel lactylation modification of SPTAN1 in HCC, revealing an unrecognized mechanism through which SPTAN1 contributes to oncogenesis. Specifically, we identified that SPTAN1 undergoes lactylation at the K1952 and K1957 residues in HCC, which is caused by HK2‐induced lactate production promoted by HBV. HBV‐induced formation of SPTAN1‐kla disrupted the LLPS of cytoplasmic SPTAN1, facilitating its translocation to the nucleus. Within the nucleus, SPTAN1‐kla exhibited a dual oncogenic function. It promoted HCC cell proliferation by interacting with CBFB to activate the NOTCH1/HES1 signaling pathway, highlighting a novel mechanism by which SPTAN1‐kla influences cellular proliferation within the tumor microenvironment. Additionally, SPTAN1‐kla enhanced the functional inhibition of CD8^+^ T cells by increasing the activity of the COX2/mPGES1/PGE2 pathway. This immunomodulatory effect contributes to the immune evasion of HCC cells, thereby exacerbating tumor progression.

To date, the role of SPTAN1 in tumors has mostly been defined as a tumor marker or potential therapeutic target.^[^ ^21^ ^]^ Given SPTAN1's function as a skeletal protein crucial for maintaining cell shape and cytoskeletal integrity, and its involvement in DNA repair,^[^ ^22^ ^]^ its presence in the nuclei of tumor cells may substantially influence tumor progression. Notably, our findings indicate that HBV can promote the occurrence of SPTAN1‐kla in HCC cell nuclei. Previous research has identified enzymes associated with kla, including P300,^[^ ^23^ ^]^ HDAC1‐3,^[^ ^24^, ^25^ ^]^ AARS1,^[^ ^26^ ^]^ and AARS2.^[^ ^27^ ^]^ In this study, we have elucidated the specific enzymes associated with SPTAN1‐kla, highlighting the role of AARS1 in promoting its formation and HDAC1 in inhibiting it.

## 4 Conclusion 

Our findings are particularly significant in the context of HBV‐related HCC, where SPTAN1‐kla emerges as a potential therapeutic target. These findings not only expand our understanding of the complex mechanisms underlying HCC tumorigenesis but also pave the way for the development of targeted therapies aimed at disrupting the oncogenic functions of SPTAN1‐kla.

## 5 Experimental Section

### Reagents and Kits

Aminoacyl tRNA synthetase‐IN‐1 (CAS: HY‐108939), PGE2 (CAS: HY‐101952), OMP 52M51 (CAS: HY‐P99258), Tangeretin (CAS: HY‐N0133) and SC‐58125 (CAS: HY‐W013164) were obtained from MedChemExpress LLC. Diethylnitrosamine (DEN) (CAS: 55‐18‐5) was obtained from Shanghai Acmec Biochemical Co., Ltd. The sources of other compounds and reagents are as follows: Collagenase from Clostridium histolyticum, type IV (Collagenase IV) (Sigma‐Aldrich, CAS: C5138), Deoxyribonuclease I from bovine pancreas (DNase I) (Sigma‐Aldrich, CAS: DN25), Ficoll Plus separation buffer (Solarbio, CAS: P4350), Cell Counting Kit‐8 (Beyotime, CAS: C0038), Tumor Dissociation Kit (Miltenyi, CAS: 130‐096‐730), Cell Staining Buffer (Biolegend, CAS: 420 201), Phosphate‐buffered saline (PBS) (Solarbio, CAS: P1020), One‐step TUNEL in situ apoptosis kit (Green, FITC) (Elabscience, CAS: E‐CK‐A320), Mouse PGE2 ELISA Kit (MultiSciences Biotech, Hangzhou, EK8103).

### Human Liver Samples

The HCC tissue samples (Table S4, Supporting Information) utilized in this study were provided by the Fujian Medical University Union Hospital. All research was conducted per the Declarations of Helsinki and Istanbul. All research was approved by the Institutional Ethics Committee of Fujian Medical University (Approval number: 2024‐014), and written consent was obtained in writing by all subjects. The tumor stage and grade were assessed according to the eighth edition of the American Joint Committee on Cancer (AJCC) criteria, and a histopathological examination confirmed the diagnosis of HCC.

### Animals

Male C57BL/6J mice, aged 4 to 6 weeks and weighing ≈17–22 grams, were obtained from Shanghai SLAC Laboratory Animal Co., Ltd. These mice were housed under specific pathogen‐free (SPF) conditions, with the temperature kept within the 21–23 °C range and the humidity maintained between 40–55%. All mice were subsequently sacrificed when they reached ethical endpoints. All animal experiments were approved by the Experimental Animal Ethics Committee of Fujian Medical University (IACUC FJMU 2024‐0328).

To establish a subcutaneous tumor model, 4‐week‐old male C57BL/6J mice were randomly allocated to various experimental groups. Subsequently, 1 × 10^7^ cells were injected subcutaneously into the right flank of each mouse. Following a period of 20 days to allow for tumor growth, the tumor tissues were harvested.

Construction of HBV^−^HCC and HBV^+^HCC models. We have successfully established a primary HCC model in our previous research.^[^ ^9^ ^]^ On this basis, 4‐week‐old male C57BL/6N mice were randomly divided into a control group (HBV^−^HCC) and an HBV infection model group (HBV^+^HCC), with 6 mice in each group. The HBV^−^HCC group refers to mice receiving intraperitoneal injection of Diethylnitrosamine (DEN, 50 mg kg^−1^) once every 2 weeks for 32 consecutive weeks. The HBV^+^HCC group received intravenous injection of rAAV8‐1.3 HBV virus, based on the HCC group, and mice were euthanized under anesthesia after 4 weeks. In addition, the hydrodynamic injection (HDI) of HBV was performed with the pAAV HBV plasmid. The liver function of mice (AST/ALT, A/G, ALP, TBIL and DBIL) and five items of hepatitis B (HBsAg, HBsAb, HBeAg, HBeAb and HBcAb) were analyzed.

Three peptides targeting the K1952 and K1957 sites were purchased from Anhui Guoping Pharmaceutical Co., Ltd. The peptide sequences are: Peptide_1: HLYVSPWGG‐EENISSKM‐(lactyl)K‐GLNG‐(lactyl)K‐V; Peptide_2: HLYVSPWGG‐NISSKM‐(lactyl)K‐GLNG‐(lactyl)K‐VSD; Peptide_3: HLYVSPWGG‐SSKM‐(lactyl)K‐GLNG‐(lactyl)K‐VSDLE. We used intraperitoneal injection of peptides (5 mg kg^−1^) thrice a week for two weeks.

### Plasmids

Plasmids Myc‐SPTAN1, Myc‐SPTAN1‐K1952R, Myc‐SPTAN1‐K1957R, Myc‐SPTAN1‐(K1952R + K1957R), GFP‐SPTAN1, GFP‐SPTAN1‐MU, Flag‐CBFB, Flag‐AARS1, Flag‐HDAC1, and Flag‐HDAC2 were obtained from Tsingke Biotechnology Co., Ltd.

### Cell Lines

The human hepatocellular carcinoma cell lines Hep3B, Huh7, HepG2.2.15, and the murine HCC cell lines H22, Hepa1‐6 were all cultured in Dulbecco's Modified Eagle Medium (DMEM) enriched with 10% fetal bovine serum (FBS) and 100 units/ml of penicillin/streptomycin. HepaRG cells were maintained in RPMI 1640 containing 10% FBS and 100 units mL^−1^ of penicillin/streptomycin. HepAD38 cells were stored in DMEM containing 10% FBS, 1% sodium pyruvate, 3 µg mL^−1^ doxycycline, and 400 µg mL^−1^ G418. The HBV genome was subcloned into the pcDNA3.1 expression vector to generate the pcDNA‐HBV construct. Huh7 cells were then stably transfected with this plasmid using Lipofectamine 3000 reagent (Invitrogen) according to the manufacturer's recommended protocol. Following transfection, cells were cultured in selection medium containing 400 µg mL^−1^ G418 to establish clonal cell populations with sustained HBV replication. These cells were maintained at 37 °C in a 5% CO_2_ incubator to guarantee consistent growth conditions. Routine mycoplasma contamination testing was conducted on the cell lines, confirming their absence of mycoplasma contamination.

To construct SPTAN1 or NOTCH1 knockout cell lines, the sgRNA (GTTCCTTGAAGCGGTGGTAT) targeting SPTAN1 and the sgRNA (CGTTGACGTCGATCTCGCATCGG) targeting NOTCH1 were designed, and CRISPRv2 gRNA lentiviral plasmids were subsequently constructed. These plasmids were combined with CRISPR/Cas9 technology to transfect the gRNAs into Hep3B and Huh7 cells. Using 293T cells for lentivirus packaging, Hep3B and Huh7 cells were infected with the virus. After a 5‐day selection period using puromycin (3 µg mL^−1^), the cell lines (Hep3B/sg/SPTAN1, Huh7/sg/SPTAN1, Hep3B/sg/NOTCH1, and Huh7/sg/NOTCH1) were successfully established.

### shRNA Transfection

The shRNA targeting human AARS1 and HDAC1 was obtained from Tsingke Biotechnology Co., Ltd. The shRNAs were transfected into cells using lipofectamine 3000. After 48 h, immunoprecipitation was used to detect the protein expression levels of SPTAN1‐kla.

### siRNA Transfection

siRNA targeting human NOTCH1, HES1, HK1, HK2, LDHA, PKM, TPI1, and SCL16A4 were obtained from GenePharma Corporation (Shanghai, China). According to the instructions, we introduced an appropriate amount of siRNA into cells through transfection reagents. After 36 h, Western blotting was used to detect the protein expression.

### TUNEL Staining

Tissue TUNEL detection was performed using the TUNEL assay kit according to the manufacturer's instructions. In short, paraffin sections of mouse tumors were subjected to dewaxing treatment, followed by incubation at room temperature with terminal deoxynucleotidyltransferase (TdT) equilibration working buffer for 30 min, followed by incubation at 37 °C with TdT enzyme working solution for 60 min. Then, the cell nucleus was stained with DAPI. Images were obtained using a fluorescence microscope (Axiolmager. D2, ZEISS, Germany).

### CCK8 Assay

Cell viability was evaluated using the Cell Counting Kit‐8 (CCK‐8) assay. HCC cells were enzymatically dissociated using trypsin, centrifuged, and resuspended in complete culture medium to establish a cell suspension with a concentration of ≈5 × 10^3^ cells per well in 96‐well microplates. Following treatments, 10 µL of CCK‐8 reagent was added to each well, and plates were incubated for 2 h at 37 °C under standard cell culture conditions (5% CO_2_, humidified atmosphere). Absorbance was measured at 450 nm using a microplate reader (BioTek, Winooski, VT).

Additional methods were listed in the supplemental information.

## Conflict of Interest

The authors declare no conflict of interest.

## Author Contributions

Z.W., D.Y., and L.W. contributed equally to this work. Z.W. performed conception and design; Z.W., D.Y., and L.W. performed investigation and data curation; J.L., B.P., Z.Z., X.Z., and Y.Y. performed development of methodology and validation; Z.W. and N.T. wrote and reviewed the manuscript; Z.W. and N.T. acquired funding; N.T. performed study supervision.

## Supporting information



Supporting Information

## Data Availability

The data that support the findings of this study are available from the corresponding author upon reasonable request.
