# Supplementary material for: Lactylated SPTAN1 Accelerates Hepatocellular Carcinoma Progression by Promoting NOTCH1/HES1 Activation and Immunosuppression
Source: Adv Sci (Weinh). 2025 Nov 16;13(6):e07068. doi: 10.1002/advs.202507068 (PMC12866801; doi:10.1002/advs.202507068)
Supplement: Supplementary file 1 — Supporting Information [file ADVS-13-e07068-s001.docx]

**Lactylated SPTAN1 Accelerates Hepatocellular Carcinoma Progression by Promoting NOTCH1/HES1 Activation and Immunosuppression**

**Contents**

Supplementary materials and methods……………………………………………...02

Supplementary Figures…………………………………………………………….. 07

Supplementary Tables…………………………………………………………........16

**Supplementary materials and methods**

**Immunoprecipitation and immunoblotting**

Cells were lysed in RIPA buffer (composed of 50 mM Tris-HCl at pH 7.4, 150 mM NaCl, 1 mM EDTA, 1% NP-40, 0.25% NaDoc, and 10% glycerol) supplemented with phenylmethylsulfonyl fluoride (PMSF; Solarbio, P8340) and a protease inhibitor cocktail (1:100; Sigma-Aldrich, P8340) for 15 minutes at 4°C. Five percent of the total lysates were reserved as input for each sample. The remaining lysate was incubated with 1 μg of primary antibody on a rotator at 4°C overnight. Subsequently, Protein G sepharose was added and incubated for an additional 4 hours at 4°C. The Protein G sepharose-bound complexes were resolved using SDS-PAGE gels and transferred to PVDF membranes. Immunoblotting of both the cell lysates and immunoprecipitates was carried out using the specified primary antibodies overnight at 4°C, followed by incubation with secondary antibodies for 1 hour at room temperature. The bands were detected and visualized using a Hypersensitive ECL Chemiluminescence Kit sourced from ABP Biosciences (Beltsville, MD). The antibodies used are as follows: anti-L-Lactyl Lysine (PTM-1401RM, PTM Biolabs Inc); anti-HA-Tag (AE008, Abclonal); anti-DDDDK-Tag (AE005, Abclonal); anti-SPTAN1(sc-376849, Santa Cruz Biotechnology); anti-GAPDH (2118, Cell Signaling Technology); anti-Myc-Tag (2276, Cell Signaling Technology); anti-β-Tubulin (2146, Cell Signaling Technology); anti-CDK4 (12790, Cell Signaling Technology); anti-NOTCH1 (20687-1-AP, Proteintech), anti-mPGES1 (ab62050, abcam), anti-COX2 (12375-1-AP, Proteintech), anti-HES1 (DF7569, Affinity) and CD8α (85336, Cell Signaling Technology). The antibody ratio used for Co-IP is 1:50. The ratio of antibodies used for immunoblotting is 1:1000. The ratio of all secondary antibodies is 1:5000.

**Nuclear and cytoplasmic extraction**

The nuclear and cytoplasmic protein extraction kit from Beyotime (Wuhan, China) was employed to carry out nuclear and cytoplasmic extraction, adhering strictly to the manufacturer's instructions. The resultant fractions were then subjected to Co-IP and immunoblotting analysis.

**Purification of GFP-SPTAN1-MU protein**

The protein sequence of GFP-SPTAN1-MU, tailored for optimal codon usage in *E. coli*, was fused with an N-terminal polyhistidine tag and subsequently cloned into the pET32a vector. The recombinant construct was then expressed in *Escherichia coli BL21* (DE3) cells, which were cultivated until they reached an OD600 value ranging from 0.4 to 0.6. To enhance protein expression, an optimal concentration of IPTG inducer was administered. The cells were further incubated in a shaker at 37°C and 200 rpm for a duration of 6 to 8 hours. Subsequently, the protein was purified utilizing a Ni-NTA column in a buffer system composed of 20 mmol/L Tris HCl, 500 mmol/L NaCl, 1 mmol/L DTT, and 10% glycerol. The purified protein was then filtered and concentrated to achieve a final concentration of 20 mmol/L.

***In vitro* detection of LLPS**

The purified GFP-SPTAN1-MU protein was diluted to the desired concentration in a buffer consisting of 20 mmol/L Tris HCl, 75 mmol/L KCl, and 1 mmol/L DTT. The mixture was then incubated at 4°C for a period of 5 to 10 minutes to facilitate phase separation. Following this, 20 microliters of the turbid solution were injected into a pre-prepared imaging chamber. Imaging was subsequently performed using a Leica SP8 confocal microscope to observe and analyze the phase separation phenomenon.

**Label-free proteomics analysis and quantitative lactylproteomics analysis**

The label-free proteomics analysis and quantitative lactylproteomics analysis were provided by Shanghai Luming Biological Technology co.Ltd.

**Detection and analysis of DNA promoter methylation in HES1**

The detection and analysis of DNA promoter methylation in HES1 were conducted by Genesky Biotechnologies Inc., Shanghai. The obtained “DNA” samples were subjected to bisulfite treatment. Subsequently, a PCR approach was employed to amplify the fragment upstream (1000 bp) of the HES1 gene promoter using the following primer sequences: *Hes1*-F: GTTTAAGAAAAGGGGGGTTAGAGGAG, and *Hes1*-R: TCTTCCAAATCCCATCACAAAAAC. The PCR products were purified using Shrimp Alkaline Phosphatase (SAP) from Promega and Exonuclease I (EXO I) from Epicentre, followed by sequencing using the BigDye 3.1 kit from ABI. The sequencing reactions were purified with alcohol, loaded onto an ABI 3730 sequencer, and the results were analyzed.

**NOTCH1 gene methylation detection**

Amplification reaction process: pre-denaturation (95℃, 3 min); denaturation (95℃, 15s), annealing (60℃, 15s), extension (72℃, 60 s), a total of 35 cycles were performed; extension (72℃, 5 min). The primer sequence for NOTCH1 methylation specific PCR (MSP) was Forward, 5'-GTTTCGTTTTTTTATTTCGTTC-3'; Reverse, 5'-GAACGCTCTACTACTTCTCGTT-3' (product length 167bp). Unmethylated primer sequence: Forward, 5'-GTTTTTTTTTTTTATTTTGTTG-3'; Reverse, 5'-CAACACCTAAAACTACTTCTCATT-3' (product length 168bp).

**Dual-luciferase reporter assay (DLR)**

The pGL3-NOTCH1 promoter-Luc reporter plasmid, derived from human sources, was acquired from Tsingke Biotechnology Co. Ltd. Subsequently, cells were co-transfected with this reporter plasmid, along with Flag-CBFB, Myc-SPTAN1-MU, Myc-SPTAN1-kla, and the pGL3-basic vector, utilizing lipofectamine 3000 as the transfection reagent. As an internal control, pRL-TK (supplied by Promega) was also included in the transfection mix. Following a thirty-six-hour incubation period post-transfection, cell lysates were prepared and subjected to a dual-luciferase assay, adhering strictly to the manufacturer's guidelines provided by Promega.

**CUT&Tag**

The sample processing, sequencing, and analysis of CUT&Tag were performed by Novogene Co., Ltd. To eliminate the influence of endogenous SPTAN1 in cells, Myc-SPTAN1-MU and Myc-SPTAN1-kla plasmids were transfected into Hep3B/sg/SPTAN1 and Huh7/sg/SPTAN1 cells, respectively. After 72 hours, the cells were collected and processed according to the following steps: binding the cells to magnetic beads, incubation with primary antibody (Myc), incubation with secondary antibody, pA/G-Tn5, fragmentation, DNA extraction, and PCR amplification. The obtained Raw data was compared, peak identified, motif analyzed and enriched, peak analyzed and released, differential peak related gene identified, and KEGG enriched.

**RNA-seq**

Novogene Co., Ltd. conducted the entire RNA-seq process, encompassing RNA extraction, quality inspection, sequencing library construction, machine sequencing, and subsequent data analysis. The data preprocessing steps involved in this process included gene differential expression analysis, as well as GO (Gene Ontology) and KEGG (Kyoto Encyclopedia of Genes and Genomes) enrichment analysis of the differentially expressed genes.

**Metabolomics analysis**

Untargeted metabolomics was detected and analyzed by Metware Biotechnology Co., Ltd. Cell samples of Class I, stored at -80°C, were thawed on ice and mixed with 500 μL of a 4:1 methanol-water solution containing an internal standard, followed by three freeze-thaw cycles involving liquid nitrogen and dry ice to ensure cell lysis. After centrifuging at 12000 rpm for 10 minutes at 4°C, 300 μL of supernatant was collected and chilled at -20°C for 30 minutes, followed by another centrifugation step. Subsequently, 200 μL aliquots of the supernatant were prepared for LC-MS analysis. Two LC/MS methods were employed: one using positive ion conditions with elution from a T3 column using a gradient of 0.1% formic acid in water and acetonitrile, and another using negative ion conditions with the same elution gradient. The column temperature was set at 40°C, the flow rate at 0.4 mL/min, and the injection volume at 4 μL. MS conditions were set using the information-dependent acquisition mode with specific source parameters and TOF MS and product ion scan parameters, including mass ranges, accumulation times, collision energies, and resolution settings, allowing for the acquisition of detailed metabolic profiles from the cell samples.

**Flow Cytometry (FCM)**

Tumor tissues were enzymatically digested using a tumor dissociation kit to isolate lymphocytes. Red blood cells were removed by adding 2 mL of Red Blood Cell Lysis Buffer, followed by a 15-minute incubation at room temperature. The resulting suspension was layered over 30 mL of Ficoll Plus and centrifuged at 900×g for 30 minutes at room temperature to isolate the lymphocyte layer. Cells were resuspended in Cell Staining Buffer at a final concentration of 2×10⁵/mL. Fluorescently labeled primary antibodies were added and incubated in the dark at 4°C for 30 minutes. Flow cytometric analysis was performed using a FACS Celesta™ Flow Cytometer (BD Biosciences, NJ, USA) to detect surface marker expression. The following antibodies used: 7-AAD (BD Biosciences, San Jose, CA), BV605-Fixable Viability Stain 575 V (BD Pharmingen), Brilliant Violet 421™ anti-mouse F4/80 (BM8, BioLegend, San Diego, CA), PE anti-mouse CD206 (C068C2, BioLegend), FITC-CD45 (QA17A26, BioLegend), APC/Cyanine7 anti-mouse CD8 (53-5.8, BioLegend), Brilliant Violet 650™ anti-mouse PD-1 (29F.1A12, BioLegend), APC-CD3 (17A2, BioLegend) and TruStain FcX™ (anti-mouse CD16/32) antibody (BioLegend). The expression of target proteins on lymphocytes was detected using a FACS Celesta™ Flow Cytometer (BD Biosciences, NJ, USA).

**Multiparametric immunofluorescence (mIF) staining**

mIF was conducted using a four-color multiple fluorescence immunohistochemical staining kit (RS0035, Immunoway, TX) that employs the tyramide signal amplification (TSA) technique. The staining process was carried out in strict accordance with the manufacturer's instructions. Subsequently, the stained images were meticulously captured using a fluorescence microscope.

**Enzyme-linked immunosorbent assay (ELISA)**

A competitive enzyme-linked immunosorbent assay (ELISA) quantified prostaglandin E2 (PGE2). Microplates pre-coated with rabbit anti-mouse antibodies were incubated with PGE2-specific monoclonal antibodies. After washing, biotinylated PGE2 tracer and samples/standards competed for binding sites. Unbound components were removed, and streptavidin-HRP conjugate was added. Following additional washes, chromogenic substrate was introduced, generating a colorimetric signal inversely proportional to PGE2 levels. The reaction was stopped, and absorbance was measured at 450 nm Standard curves enabled sample PGE2 quantification.

**Statistical analysis**

The results are presented as the mean ± SD. All statistical analyses were performed using SPSS Statistics v26.0. For normally distributed data with equal variances, t test was employed to compare two samples. For comparisons involving multiple groups, variance (ANOVA) was conducted. The graphic production was completed using GraphPad Prism V.8 software. Statistical significance was set at a *P*-value < 0.05.

**Supplementary Figures**


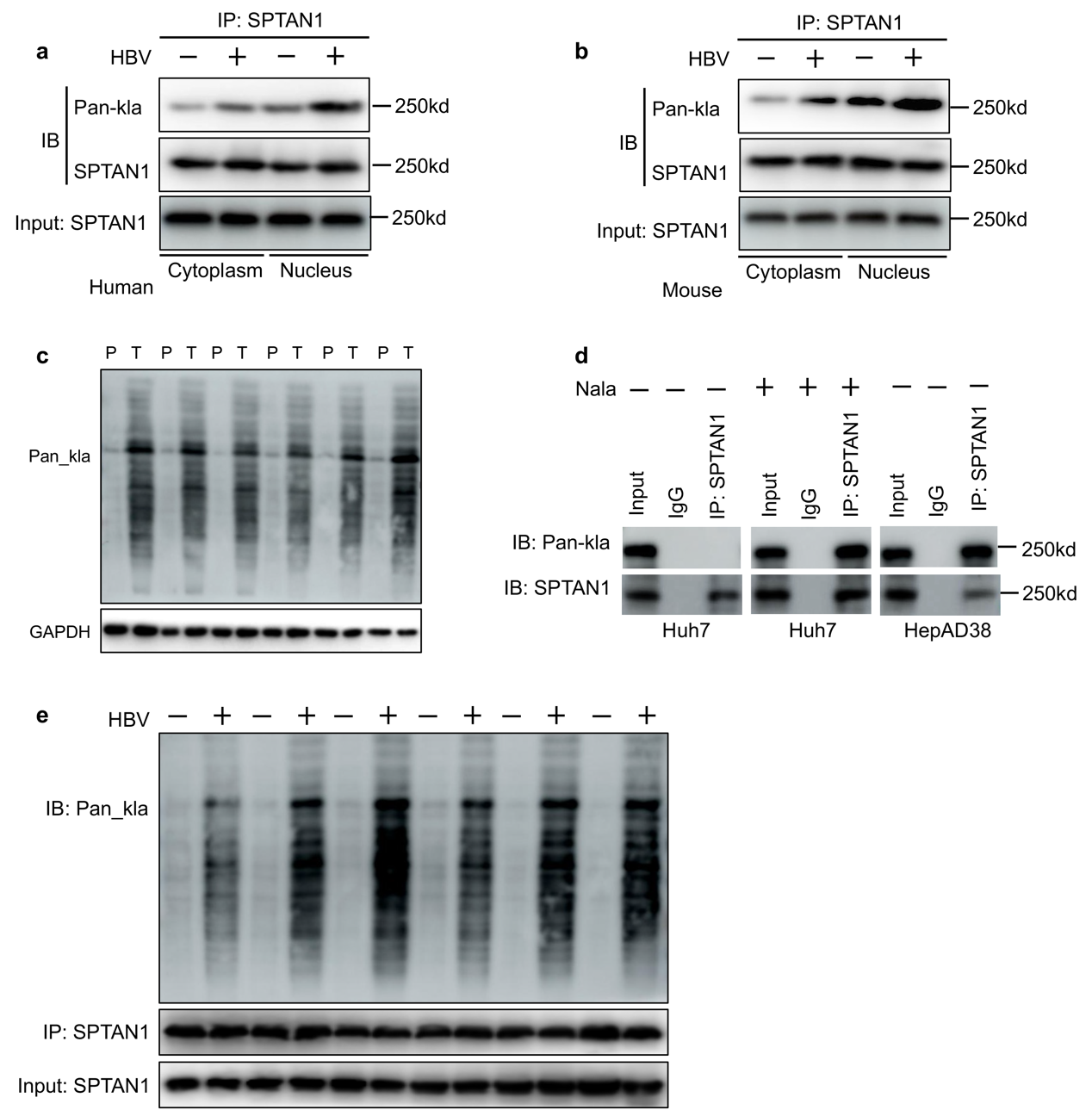


Supplementary Fig. 1 **a, b,** Co-IP detection of SPTAN1-kla levels in the nucleus and cytoplasm of HBV^+^HCC tissues in human and mice. **c,** Immunoblotting was used to analyze the Pan-kla levels in HCC tissues and adjacent tissues infected with HBV. n=128. **d**, HBV^-^HCC cells (Huh7) were treated with Nala (10 mM) for 24 hours, and SPTAN1-kla levels were detected by Co-IP assay. HBV^+^HCC cells (HepAD38) were used as positive controls. n=3. **e**, We constructed a spontaneous HCC model of HBV through hydrodynamic injection. Co-IP detection of SPTAN1-kla in the cell nucleus. n=6.


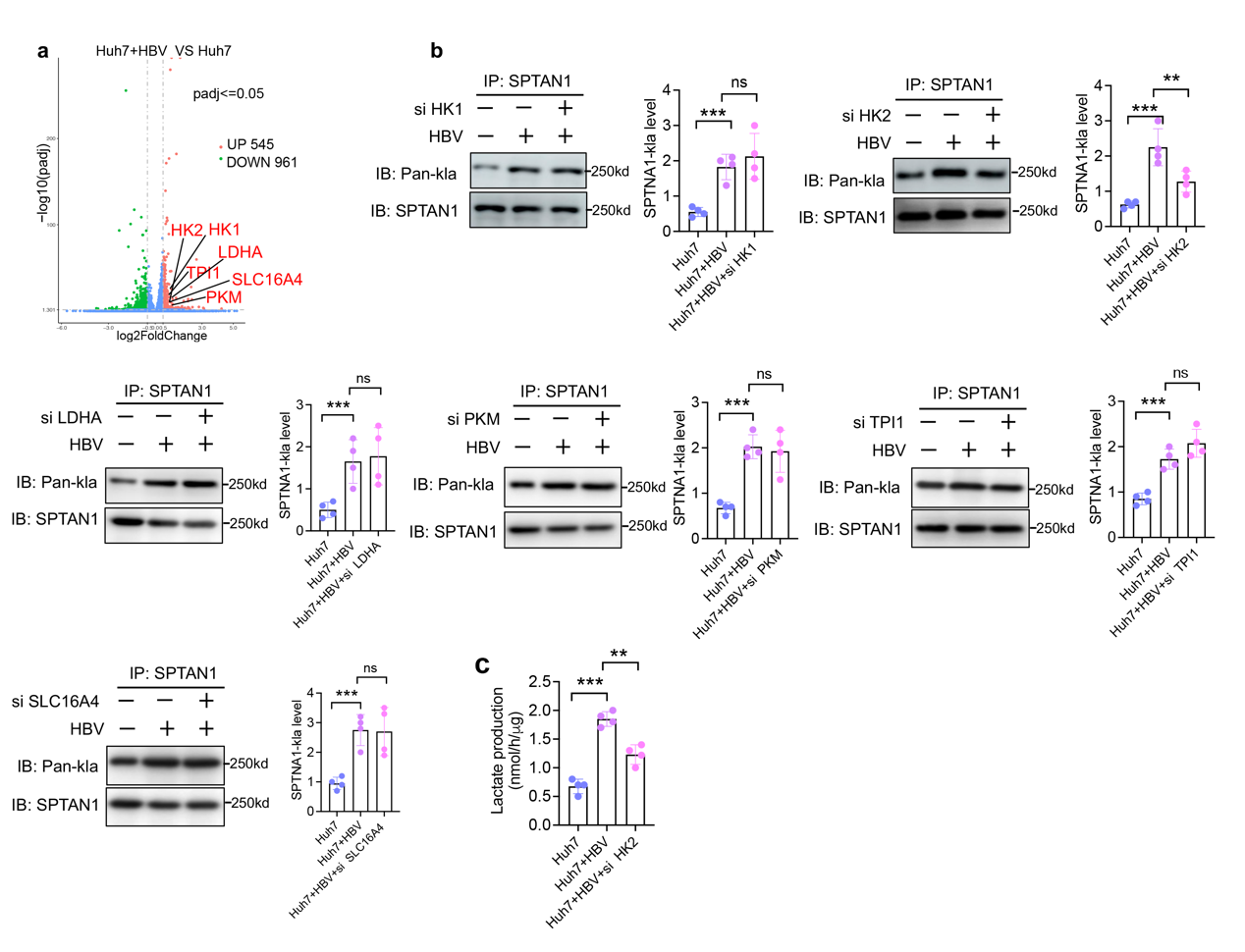


Supplementary Fig. 2 HBV induces SPTAN1-kla by promoting HK2 expression. **a,** After HBV infection of Huh7 cells, the differentially expressed genes were analyzed by RNA-seq. **b**, si RNA was used to target HK1, HK2, LDHA, PKM, TPI1 and SLC16A4 respectively. Co-IP was used to analyze the effect of si RNA on the expression level of SPTAN1-kla. n=4. Data were mean ± SD and analyzed by one-way ANOVA. **c**, Analyze the lactate concentration in the supernatant of cells. n=4. Data were mean ± SD and analyzed by one-way ANOVA. “ns” indicates no significant difference. ***P* < 0.01, ****P* < 0.001.


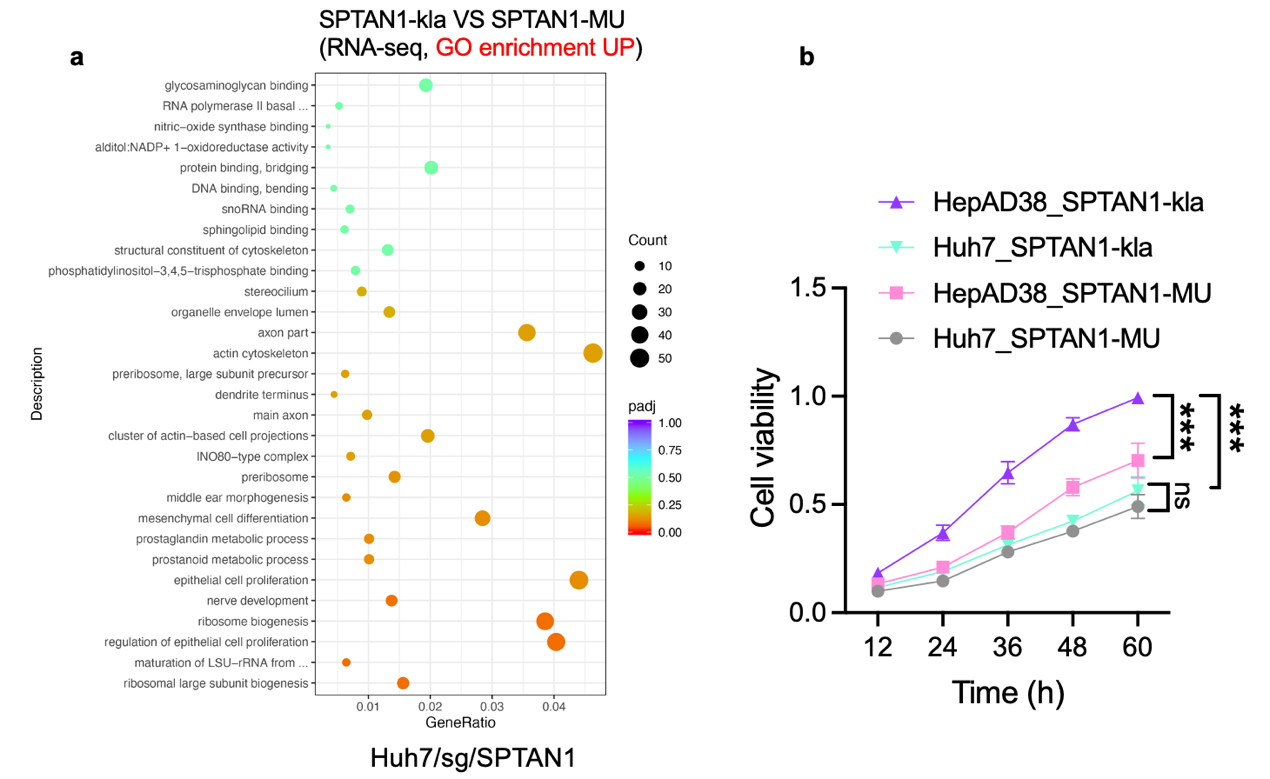


Supplementary Fig. 3 **a,** RNA-seq was used to detect differentially expressed genes in Huh7/sg/SPTAN1 cells transfected with SPTAN1-kla and SPTAN1-MU, and GO enrichment analysis was performed. **b**, The CCK-8 experiment was used to detect the viability of Huh7 cells and HepAD38 cells transfected with SPTAN1-kla or SPTAN1-MU. n=6. Data were mean ± SD and analyzed by two-way ANOVA. “ns” indicates no significant difference. ****P* < 0.001.


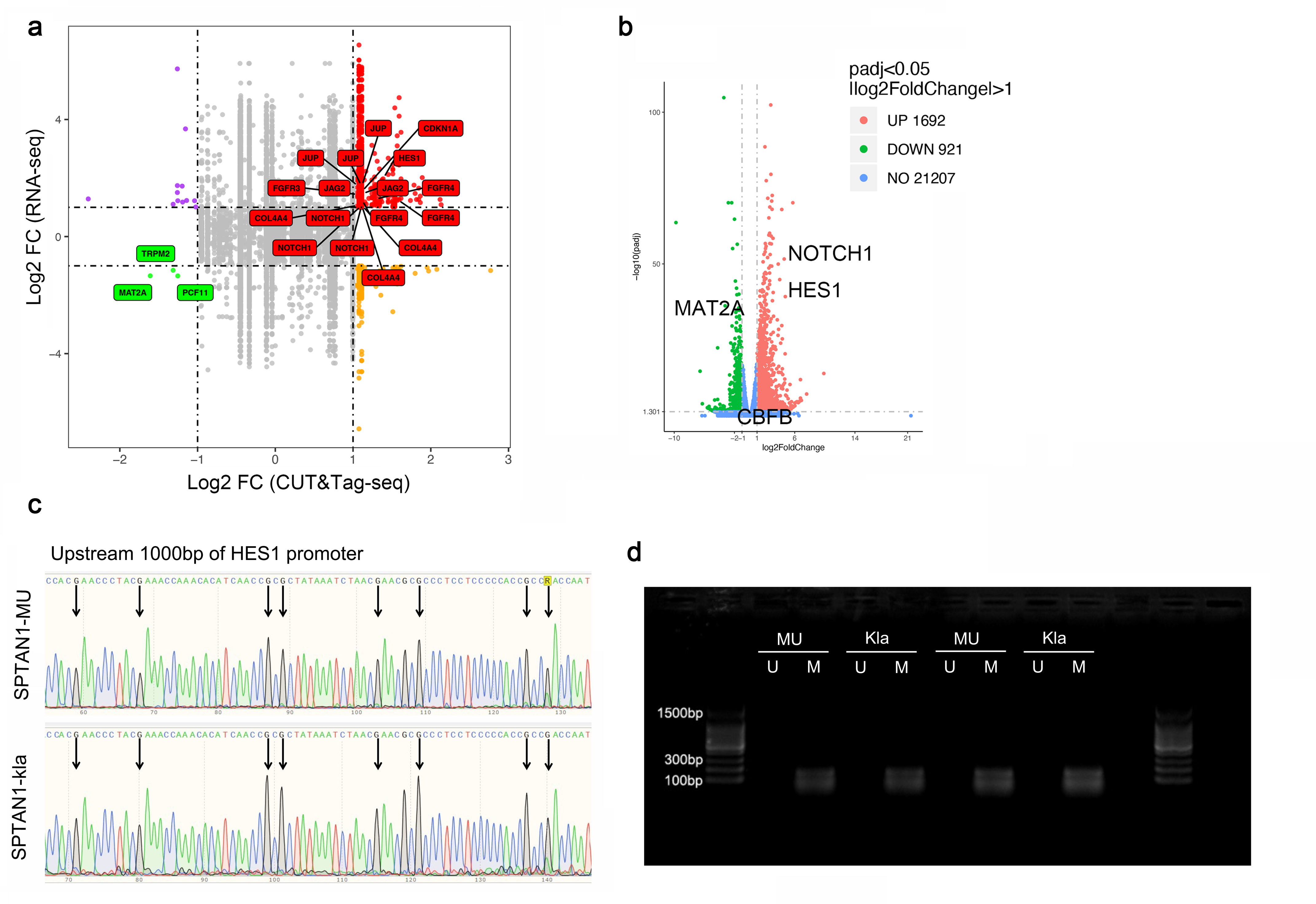


Supplementary Fig. 4 SPTAN1-kla does not affect the promoter methylation of NOTCH1 and HES1. **a,** The nine quadrant plot of CUT & Tag and RNA-seq combined analysis showed a correlation between the differences in epigenetic and expression levels. **b,** The volcano map displayed the differentially expressed genes between the SPTAN1-kla and SPTAN1-MU groups in Hep3B/sg/SPTAN1 cells. **c,** CpG sites sequencing analysis of HES1 after transfection of Hep3B/sg/SPTAN1 cells with SPTAN1-MU and SPTAN1-kla. **d,** NOTCH1 gene methylation detection.

**
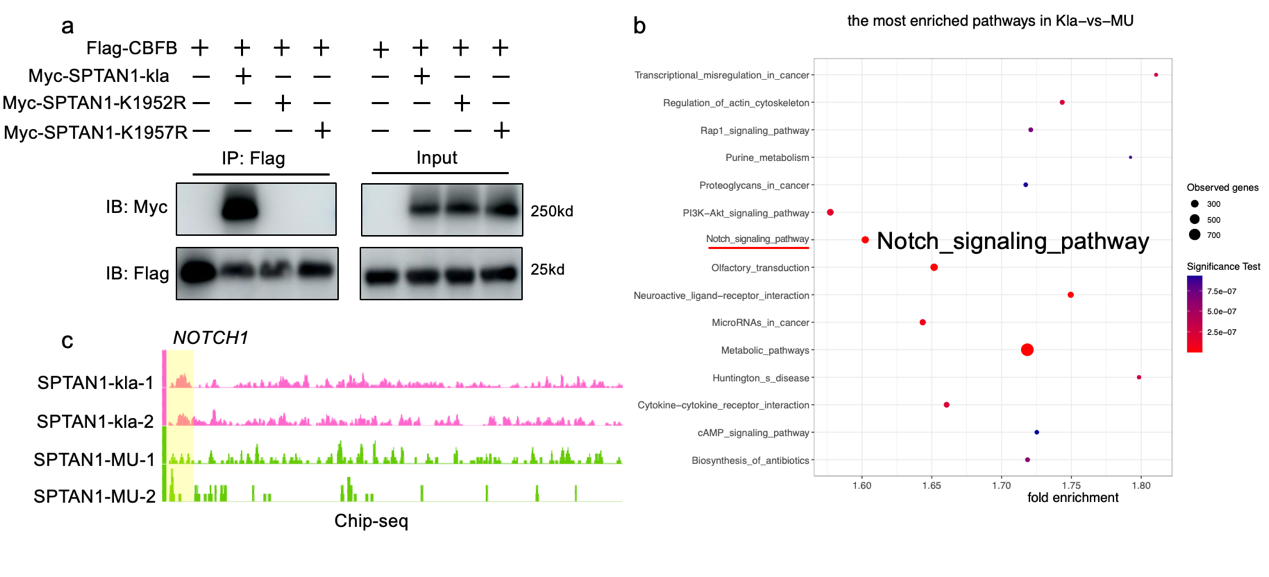
**

Supplementary Fig. 5 SPTAN1-kla promotes CBFB binding to NOTCH1 promoter. **a,** Co-IP verification of the interaction between SPTAN1 single site mutation and CBFB. **b,** The Kyoto Encyclopedia of Genes and Genomes (KEGG) annotation for the genes associated with peaks. **c,** Comparison of IGV results of NOTCH1 gene between SPTAN1-MU and SPTAN1-kla group (n = 2).

**
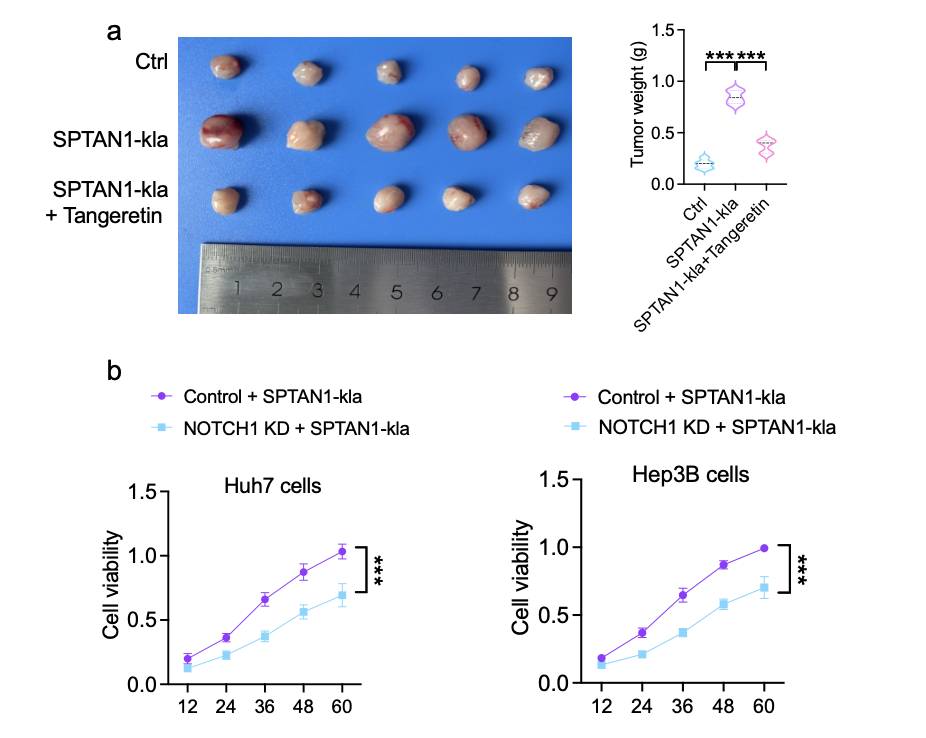
**

Supplementary Fig. 6 SPTAN1-kla promotes the progression of HCC by activating the NOTCH1/HES1 signaling pathway. **a,** Mice were intraperitoneally injected with Tangeretin (20 mg/kg) every 2 days on the 15th day of subcutaneous tumor growth, for a total of 3 injections, and then the size of the subcutaneous tumor was observed. The Ctrl group represents Hepa1-6/sg/SPTAN1 cells, the SPTAN1-kla group represents Hepa1-6/sg/SPTAN1 cells transfected with SPTAN1-kla, and the SPTAN1-kla + Tangeretin group represents mice treated with Tangeretin on the basis of the SPTAN1-kla group. n=5. Data were mean ± SD and analyzed by one-way ANOVA. **b,** NOTCH1 KD cells were subjected to the CCK-8 assay after SPTAN1-kla treatment. Cell viability was tested every 12 hours. The horizontal axis indicated that cell viability was detected every 12 hours. n=6. Data were mean ± SD and analyzed by two-way ANOVA. ****P* < 0.001.


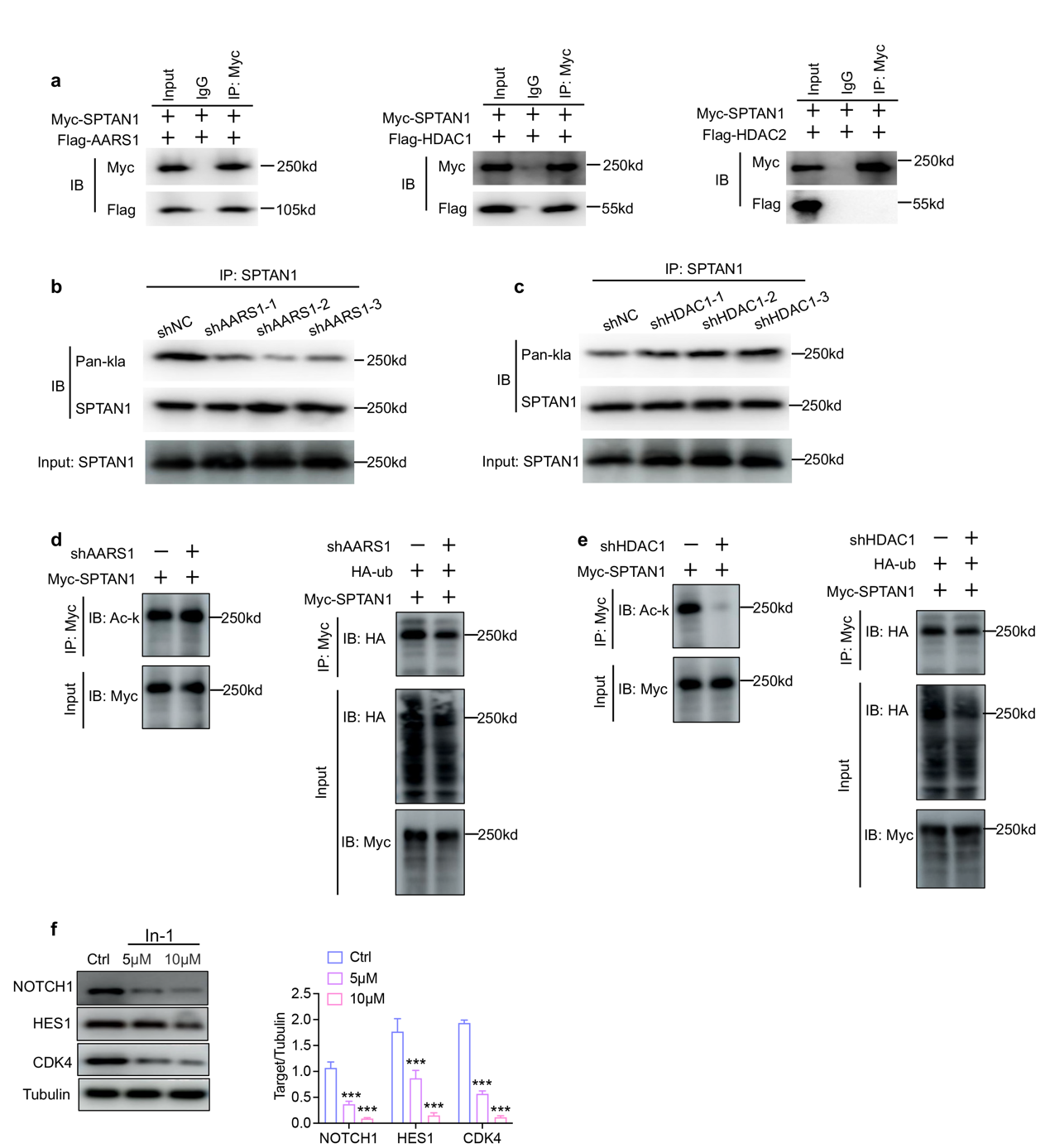


Supplementary Fig. 7 The lactylation and delactylation of SPTAN1-kla were identified. **a,** The interaction of SPTAN1 with AARS1, HDAC1 and HDAC2 was detected by Co-IP. n=3. **b,** **c,** Co-IP was used to detect the effect of targeting AARS1 and HDAC1 on the expression level of SPTAN1-kla. n=3. **d,** Co-IP analysis of the effect of shAARS1 on SPTAN1 acetylation and ubiquitination. n=3. **e,** Co-IP analysis of the effect of shHDAC1 on SPTAN1 acetylation and ubiquitination. n=3. **f,** The effect of AARS1 inhibitors (aminoacyl tRNA synthase-in-1) on NOTCH1, HES1 and CDK4 protein expression was detected by immunoblotting. n=4. Data were mean ± SD and analyzed by one-way ANOVA.

**
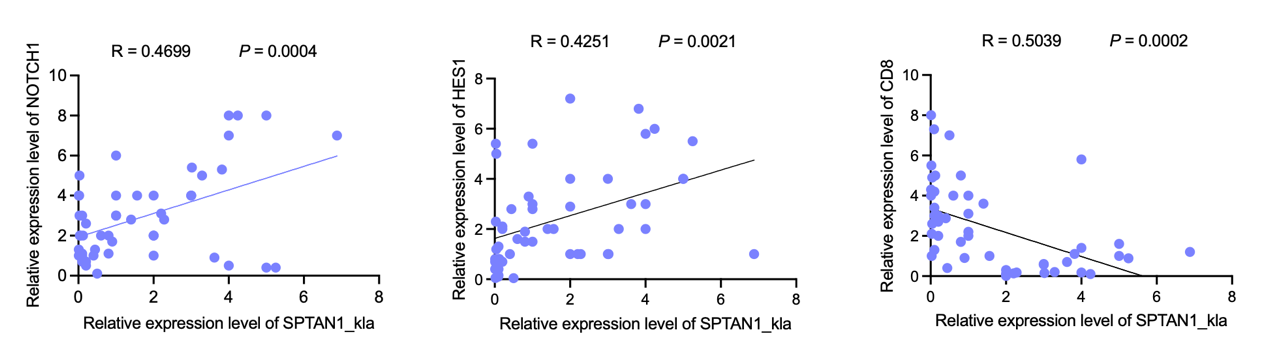
**

Supplementary Fig. 8 Correlation between SPTAN1-kla, NOTCH1, HES1 and CD8 in human HCC. Pearson correlation analysis of the correlation between SPTAN1-kla, NOTCH1, HES1 and CD8 in human HCC. n=50.

**
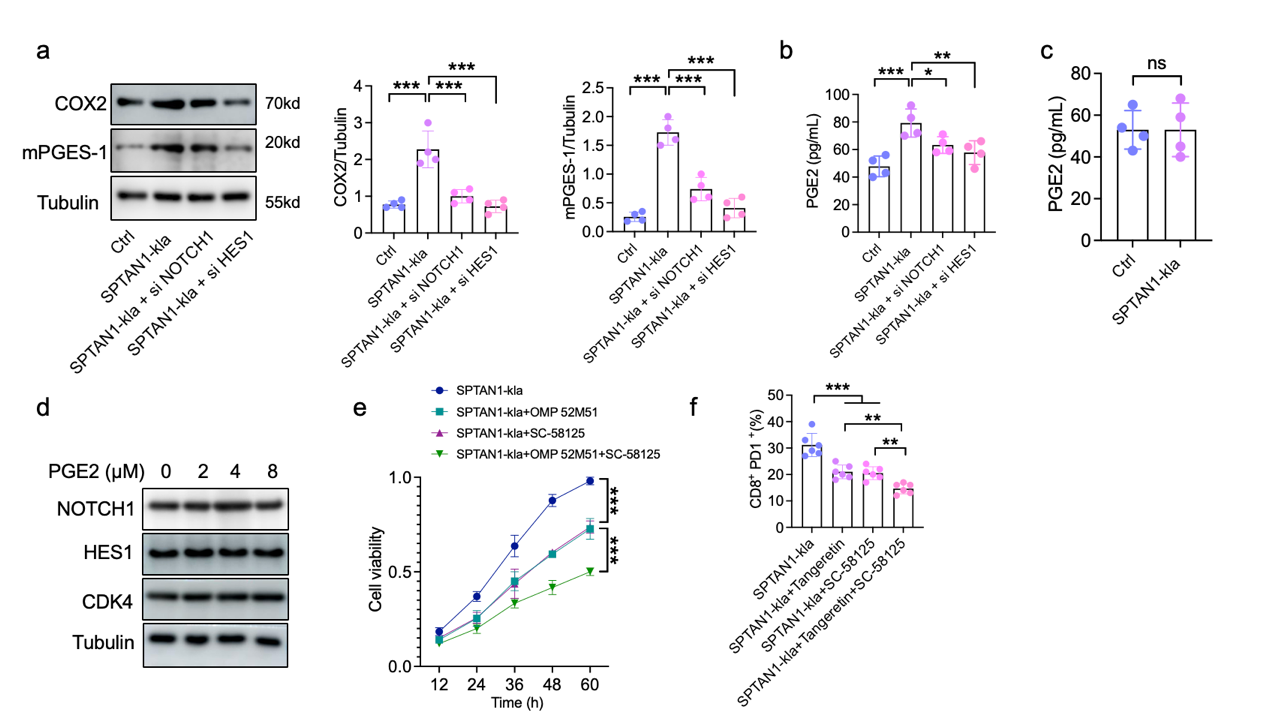
**

Supplementary Fig. 9 SPTAN1-kla promoted HCC proliferation and immune escape through the synergistic effect of the NOTCH1/HES1 and PGE2 pathways. **a,** The effect of si NOTCH1 or si HES1 on COX2 and mPGES1 protein expression was detected by immunoblotting in Hep3B/sg/SPTAN1 cells. n=4. Data were mean ± SD and analyzed by one-way ANOVA. **b,** ELISA analysis of PGE2 secretion levels in Hep3B/sg/SPTAN1 cells. n=4. Data were mean ± SD and analyzed by one-way ANOVA. **c,** ELISA analysis of PGE2 secretion levels in Hep3B/sg/NOTCH1 cells. n=4. Data were mean ± SD and analyzed by one-way ANOVA. **d,** The effect of PGE2 on NOTCH1, HES1 and CDK4 protein expression was detected by immunoblotting. **e,** Cells overexpressing SPTAN1-kla were analyzed by CCK-8 assay after combined or alone treatment with OMP 52M51(25 μg/mL) and SC-58125 (25 μmol/L) treatment. n=6. Data were mean ± SD and analyzed by two-way ANOVA. **f,** After the subcutaneous tumor model was established, the mice were treated with Tangeretin (20 mg/kg) and SC-58125 (10 mg/kg) in combination or alone. FCM analysis of changes in the proportion of CD8^+^ PD1^+^ cells. n=6. Data were mean ± SD and analyzed by one-way ANOVA.

**
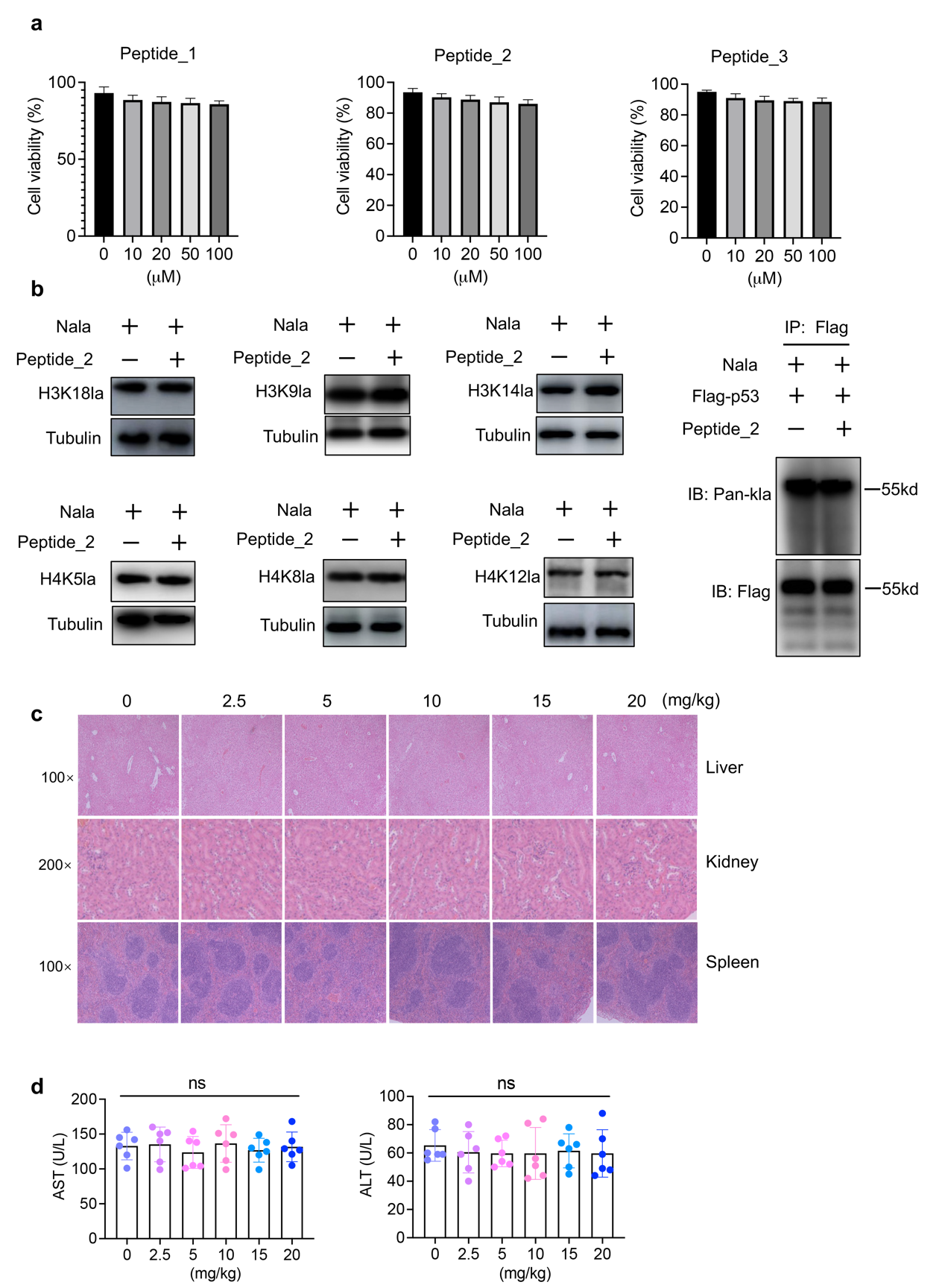
**

Supplementary Fig. 10 *In vitro* cytotoxicity and *in vivo* safety analysis of peptide_2. **a,** The viability of HepaRG cells was analyzed by CCK8 under treatment with peptides of different concentrations (0-100μM). **b,** Immunoblotting was used to analyze the effects of peptide_2 on the lactylation of histones and p53. **c,** HE staining was used to analyze the tissue structure of liver, kidney and spleen in mice treated with different concentrations of peptide_2. **d,** AST and ALT levels in mouse serum were measured. n=6. One-way ANOVA were used for statistical analysis. “ns” indicates no significant difference.


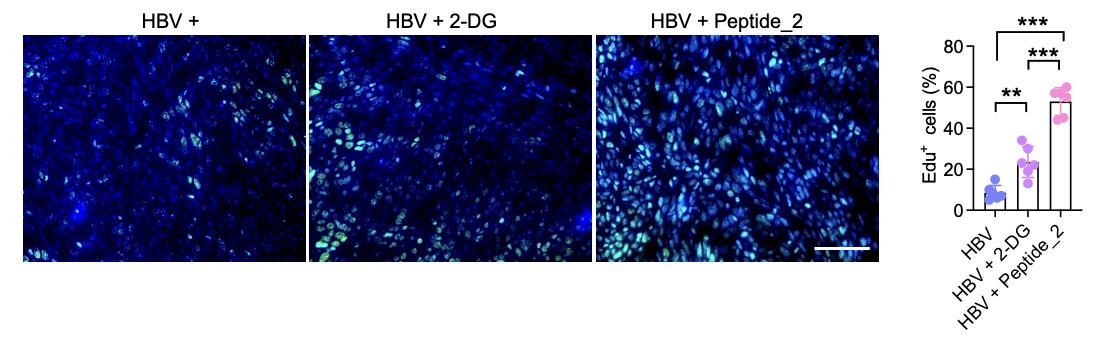


Supplementary Fig. 11 TUNEL staining for detecting apoptosis levels in subcutaneous tumors of mice. n=6. Data were mean ± SD and analyzed by one-way ANOVA. Bar=100 μm. ***P* < 0.01, ****P* < 0.001.

**
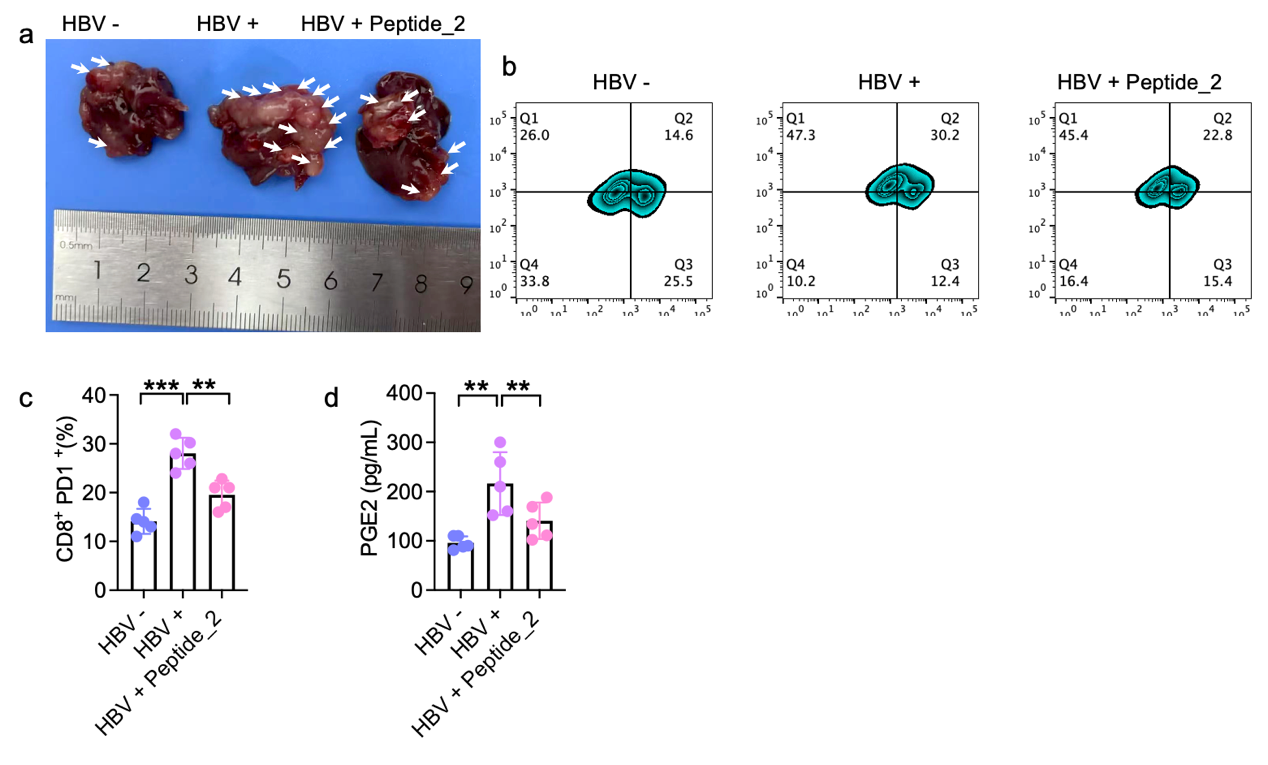
**

Supplementary Fig. 12 Analysis of the effects of peptide_2 treatment on tumor growth, CD8^+^ PD1^+^ cells infiltration, and PGE2 secretion in an orthotopic HCC model. **a,** HCC infected with HBV were used to establish an orthotopic HCC model and treated the mice with peptide_2. The white arrow marks the tumor nodule. **b, c,** FCM analysis of changes in the proportion of CD8^+^ PD1^+^ cells. n=5. Data were mean ± SD and analyzed by one-way ANOVA. **d** ELISA analysis of PGE2 secretion levels in mouse serum. n=5. Data were mean ± SD and analyzed by one-way ANOVA. ***P* < 0.01, ****P* < 0.001.

**Supplementary Tables**

**Supplementary Table 1. Liver function testing.**

| Project | HBV-_1 | HBV-_2 | HBV-_3 | HBV+_1 | HBV+_2 | HBV+_3 |
| --- | --- | --- | --- | --- | --- | --- |
| TBIL | 0.5 | 0.3 | 0.3 | 1 | 1.2 | 0.8 |
| DBIL | 0.2 | 0.2 | 0.2 | 0.3 | 4.1 | 0.3 |
| IBIL | 0.3 | 0.1 | 0.1 | 0.7 | -2.9 | 0.5 |
| TP | 8.9 | 7.9 | 8.9 | 16.9 | 15.5 | 19.4 |
| ALB | 3.3 | 2.6 | 3.3 | 11.6 | 9 | 13 |
| GLB | 5.6 | 5.3 | 5.6 | 5.3 | 6.5 | 6.4 |
| A/G | 0.59 | 0.49 | 0.59 | 2.19 | 1.38 | 2.03 |
| ALT | 4 | 1 | 465 | 6525 | 5967 | 8002 |
| AST | 16 | 15 | 786 | 4372 | 9066 | 13616 |
| AST/ALT | 4 | 15 | 1.69 | 0.67 | 1.52 | 1.7 |
| ALP | 11 | 9 | 15 | 45 | 46 | 66 |
| Cholinesterase | 697 | 645 | 562 | 211 | 224 | 290 |

**Supplementary Table 2. Five tests for hepatitis B.**

| Project | HBV-_1 | HBV-_2 | HBV-_3 | HBV+_1 | HBV+_2 | HBV+_3 |
| --- | --- | --- | --- | --- | --- | --- |
| HBsAg | 0.01 | 0 | 0 | 3.57 | 4.82 | 3.07 |
| HBsAb | 0.5 | 0 | 2 | 13.96 | 10.17 | 12.46 |
| HBeAg | 0.02 | 0 | 0 | 134.56 | 68.5 | 41.76 |
| HBeAb | 1.81 | 2.9 | 1 | 2.69 | 2 | 2 |
| HBcAb | 2.09 | 1.57 | 1.57 | 1.72 | 1.96 | 1.94 |
|  |  |  |  |  |  |  |

**Supplementary Table 3. Methylation of CpG of HES1 promoter.**

| Sample | CpG No1 | CpG No2 | CpG No3 | CpG No4 | CpG No5 | CpG No6 | CpG No7 | CpG No8 | CpG No9 |
| --- | --- | --- | --- | --- | --- | --- | --- | --- | --- |
| Hep3B/sg/SPTAN1-kla_1 | 4 | 4 | 4 | 4 | 4 | 4 | 4 | 4 | 3 |
| Hep3B/sg/SPTAN1-MU_1 | 4 | 4 | 4 | 4 | 4 | 4 | 4 | 4 | 3 |
| Hep3B/sg/SPTAN1-kla_2 | 4 | 4 | 4 | 4 | 4 | 4 | 4 | 4 | 3 |
| Hep3B/sg/SPTAN1-MU_2 | 4 | 4 | 4 | 4 | 4 | 4 | 4 | 4 | 3 |
| Hep3B/sg/SPTAN1-kla_3 | 4 | 4 | 4 | 4 | 4 | 4 | 4 | 4 | 3 |
| Hep3B/sg/SPTAN1-MU_3 | 4 | 4 | 4 | 4 | 4 | 4 | 4 | 4 | 3 |
|  |  |  |  |  |  |  |  |  |  |

**Supplementary Table 4. Clinical data of HCC patients.**

| Age | Gender | HBV | HBsAg (IU/mL) | HBsAb (mIU/mL) | HBeAg (S/C.0) | HBeAb (S/C.0) | HBcAb (S/C.0) |
| --- | --- | --- | --- | --- | --- | --- | --- |
| 48 | male | (+) | 205.98 | 0.44 | 0.36 | 0.01 | 5.37 |
| 63 | male | (+) | 103.17 | 3.43 | 0.35 | 0.02 | 4.59 |
| 55 | male | (+) | 1250 | 0 | 0.77 | 0.34 | 5.03 |
| 43 | male | (+) | 920 | 0 | 2.83 | 1.28 | 5.31 |
| 56 | male | (+) | 900 | 0.62 | 1.32 | 1.74 | 4.22 |
| 50 | male | (+) | 533 | 2.28 | 0.33 | 0.01 | 5.2 |
| 45 | female | (+) | 974 | 0 | 0.43 | 1.02 | 4.97 |
| 47 | male | (+) | 708 | 1.62 | 3.24 | 0.08 | 8.2 |
| 58 | male | (+) | 1028.6 | 2.9 | 1.01 | 0.15 | 3.88 |
| 58 | female | (+) | 689 | 0.83 | 2.09 | 1.25 | 7.8 |
| 59 | male | (+) | 936 | 2.22 | 0.66 | 1.56 | 3.48 |
| 63 | male | (+) | 707.6 | 3.21 | 0.22 | 0.31 | 4.23 |
| 42 | male | (+) | 410.3 | 0.65 | 0.28 | 1.41 | 2.88 |
| 38 | male | (+) | 557 | 0 | 0.37 | 1.09 | 1.65 |
| 70 | male | (+) | 483.3 | 0.32 | 0.75 | 1.02 | 1.41 |
| 58 | male | (-) | ~~NA~~ | ~~NA~~ | ~~NA~~ | ~~NA~~ | ~~NA~~ |
| 62 | male | (-) | ~~NA~~ | ~~NA~~ | ~~NA~~ | ~~NA~~ | ~~NA~~ |
| 68 | male | (-) | ~~NA~~ | ~~NA~~ | ~~NA~~ | ~~NA~~ | ~~NA~~ |
| 59 | male | (-) | ~~NA~~ | ~~NA~~ | ~~NA~~ | ~~NA~~ | ~~NA~~ |
| 58 | female | (-) | ~~NA~~ | ~~NA~~ | ~~NA~~ | ~~NA~~ | ~~NA~~ |
| 40 | male | (-) | ~~NA~~ | ~~NA~~ | ~~NA~~ | ~~NA~~ | ~~NA~~ |
| 39 | male | (-) | ~~NA~~ | ~~NA~~ | ~~NA~~ | ~~NA~~ | ~~NA~~ |
| 57 | male | (-) | ~~NA~~ | ~~NA~~ | ~~NA~~ | ~~NA~~ | ~~NA~~ |
| 55 | female | (-) | ~~NA~~ | ~~NA~~ | ~~NA~~ | ~~NA~~ | ~~NA~~ |
| 54 | male | (-) | ~~NA~~ | ~~NA~~ | ~~NA~~ | ~~NA~~ | ~~NA~~ |
| 58 | male | (-) | ~~NA~~ | ~~NA~~ | ~~NA~~ | ~~NA~~ | ~~NA~~ |
| 66 | female | (-) | ~~NA~~ | ~~NA~~ | ~~NA~~ | ~~NA~~ | ~~NA~~ |
| 65 | male | (-) | ~~NA~~ | ~~NA~~ | ~~NA~~ | ~~NA~~ | ~~NA~~ |
| 43 | male | (-) | ~~NA~~ | ~~NA~~ | ~~NA~~ | ~~NA~~ | ~~NA~~ |
| 50 | male | (-) | ~~NA~~ | ~~NA~~ | ~~NA~~ | ~~NA~~ | ~~NA~~ |
| 51 | female | (+) | 204 | 3.2 | 0.18 | 1 | 4.23 |
| 50 | male | (+) | 773.4 | 1 | 1.29 | 1.05 | 6.81 |
| 62 | male | (+) | 182 | 0 | 3.04 | 0.09 | 4.2 |
| 47 | female | (+) | 1033 | 3.45 | 0.38 | 1.99 | 2.01 |
| 39 | male | (+) | 1019.2 | 0 | 0.4 | 0.1 | 3.7 |
| 60 | male | (+) | 424 | 1.5 | 0.52 | 1.02 | 4.9 |
| 63 | male | (+) | 821.1 | 0.9 | 4.3 | 2.2 | 7.33 |
| 62 | male | (+) | 909 | 2 | 2 | 0.62 | 3 |
| 71 | male | (+) | 592 | 1.83 | 3.39 | 1 | 8.35 |
| 50 | male | (+) | 388 | 1.89 | 0.65 | 1.56 | 3.32 |
| 51 | male | (+) | 1012 | 1.4 | 0.78 | 0.34 | 1.88 |
| 52 | male | (+) | 481 | 0.65 | 0.31 | 2.5 | 1.1 |
| 66 | male | (+) | 861 | 0.8 | 0.3 | 1.29 | 2 |
| 44 | female | (-) | ~~NA~~ | ~~NA~~ | ~~NA~~ | ~~NA~~ | ~~NA~~ |
| 45 | male | (-) | ~~NA~~ | ~~NA~~ | ~~NA~~ | ~~NA~~ | ~~NA~~ |
| 63 | male | (-) | ~~NA~~ | ~~NA~~ | ~~NA~~ | ~~NA~~ | ~~NA~~ |
| 57 | male | (-) | ~~NA~~ | ~~NA~~ | ~~NA~~ | ~~NA~~ | ~~NA~~ |
| 67 | female | (-) | ~~NA~~ | ~~NA~~ | ~~NA~~ | ~~NA~~ | ~~NA~~ |
| 48 | male | (-) | ~~NA~~ | ~~NA~~ | ~~NA~~ | ~~NA~~ | ~~NA~~ |
| 45 | male | (-) | ~~NA~~ | ~~NA~~ | ~~NA~~ | ~~NA~~ | ~~NA~~ |
| 46 | female | (-) | ~~NA~~ | ~~NA~~ | ~~NA~~ | ~~NA~~ | ~~NA~~ |
| 36 | male | (-) | ~~NA~~ | ~~NA~~ | ~~NA~~ | ~~NA~~ | ~~NA~~ |
| 49 | male | (-) | ~~NA~~ | ~~NA~~ | ~~NA~~ | ~~NA~~ | ~~NA~~ |
| 54 | male | (-) | ~~NA~~ | ~~NA~~ | ~~NA~~ | ~~NA~~ | ~~NA~~ |
| 45 | male | (-) | ~~NA~~ | ~~NA~~ | ~~NA~~ | ~~NA~~ | ~~NA~~ |
| 55 | male | (-) | ~~NA~~ | ~~NA~~ | ~~NA~~ | ~~NA~~ | ~~NA~~ |
| 57 | female | (-) | ~~NA~~ | ~~NA~~ | ~~NA~~ | ~~NA~~ | ~~NA~~ |
| 56 | male | (-) | ~~NA~~ | ~~NA~~ | ~~NA~~ | ~~NA~~ | ~~NA~~ |
| 62 | male | (-) | ~~NA~~ | ~~NA~~ | ~~NA~~ | ~~NA~~ | ~~NA~~ |
| 59 | male | (-) | ~~NA~~ | ~~NA~~ | ~~NA~~ | ~~NA~~ | ~~NA~~ |
| 44 | male | (-) | ~~NA~~ | ~~NA~~ | ~~NA~~ | ~~NA~~ | ~~NA~~ |
| 59 | male | (-) | ~~NA~~ | ~~NA~~ | ~~NA~~ | ~~NA~~ | ~~NA~~ |
| 68 | male | (-) | ~~NA~~ | ~~NA~~ | ~~NA~~ | ~~NA~~ | ~~NA~~ |
| 60 | male | (-) | ~~NA~~ | ~~NA~~ | ~~NA~~ | ~~NA~~ | ~~NA~~ |
| 60 | male | (-) | ~~NA~~ | ~~NA~~ | ~~NA~~ | ~~NA~~ | ~~NA~~ |
| 60 | male | (-) | ~~NA~~ | ~~NA~~ | ~~NA~~ | ~~NA~~ | ~~NA~~ |
| 61 | female | (-) | ~~NA~~ | ~~NA~~ | ~~NA~~ | ~~NA~~ | ~~NA~~ |
| 45 | male | (-) | ~~NA~~ | ~~NA~~ | ~~NA~~ | ~~NA~~ | ~~NA~~ |
| 50 | male | (-) | ~~NA~~ | ~~NA~~ | ~~NA~~ | ~~NA~~ | ~~NA~~ |
| 50 | male | (-) | ~~NA~~ | ~~NA~~ | ~~NA~~ | ~~NA~~ | ~~NA~~ |
| 58 | male | (-) | ~~NA~~ | ~~NA~~ | ~~NA~~ | ~~NA~~ | ~~NA~~ |
| 59 | male | (-) | ~~NA~~ | ~~NA~~ | ~~NA~~ | ~~NA~~ | ~~NA~~ |
| 40 | female | (-) | ~~NA~~ | ~~NA~~ | ~~NA~~ | ~~NA~~ | ~~NA~~ |
| 42 | male | (-) | ~~NA~~ | ~~NA~~ | ~~NA~~ | ~~NA~~ | ~~NA~~ |
| 58 | male | (-) | ~~NA~~ | ~~NA~~ | ~~NA~~ | ~~NA~~ | ~~NA~~ |
| 58 | male | (-) | ~~NA~~ | ~~NA~~ | ~~NA~~ | ~~NA~~ | ~~NA~~ |
| 56 | male | (-) | ~~NA~~ | ~~NA~~ | ~~NA~~ | ~~NA~~ | ~~NA~~ |
| 70 | male | (-) | ~~NA~~ | ~~NA~~ | ~~NA~~ | ~~NA~~ | ~~NA~~ |
| 46 | female | (-) | ~~NA~~ | ~~NA~~ | ~~NA~~ | ~~NA~~ | ~~NA~~ |
| 68 | female | (-) | ~~NA~~ | ~~NA~~ | ~~NA~~ | ~~NA~~ | ~~NA~~ |
| 70 | male | (-) | ~~NA~~ | ~~NA~~ | ~~NA~~ | ~~NA~~ | ~~NA~~ |
| 43 | male | (-) | ~~NA~~ | ~~NA~~ | ~~NA~~ | ~~NA~~ | ~~NA~~ |
| 44 | male | (-) | ~~NA~~ | ~~NA~~ | ~~NA~~ | ~~NA~~ | ~~NA~~ |
| 47 | male | (-) | ~~NA~~ | ~~NA~~ | ~~NA~~ | ~~NA~~ | ~~NA~~ |
| 50 | male | (-) | ~~NA~~ | ~~NA~~ | ~~NA~~ | ~~NA~~ | ~~NA~~ |
| 50 | male | (-) | ~~NA~~ | ~~NA~~ | ~~NA~~ | ~~NA~~ | ~~NA~~ |
| 66 | male | (-) | ~~NA~~ | ~~NA~~ | ~~NA~~ | ~~NA~~ | ~~NA~~ |
| 66 | male | (-) | ~~NA~~ | ~~NA~~ | ~~NA~~ | ~~NA~~ | ~~NA~~ |
| 54 | male | (-) | ~~NA~~ | ~~NA~~ | ~~NA~~ | ~~NA~~ | ~~NA~~ |
| 51 | male | (-) | ~~NA~~ | ~~NA~~ | ~~NA~~ | ~~NA~~ | ~~NA~~ |
| 49 | female | (-) | ~~NA~~ | ~~NA~~ | ~~NA~~ | ~~NA~~ | ~~NA~~ |
| 38 | male | (-) | ~~NA~~ | ~~NA~~ | ~~NA~~ | ~~NA~~ | ~~NA~~ |
| 39 | male | (-) | ~~NA~~ | ~~NA~~ | ~~NA~~ | ~~NA~~ | ~~NA~~ |
| 40 | male | (-) | ~~NA~~ | ~~NA~~ | ~~NA~~ | ~~NA~~ | ~~NA~~ |
| 49 | male | (-) | ~~NA~~ | ~~NA~~ | ~~NA~~ | ~~NA~~ | ~~NA~~ |
| 58 | male | (-) | ~~NA~~ | ~~NA~~ | ~~NA~~ | ~~NA~~ | ~~NA~~ |
| 57 | female | (-) | ~~NA~~ | ~~NA~~ | ~~NA~~ | ~~NA~~ | ~~NA~~ |
| 59 | female | (-) | ~~NA~~ | ~~NA~~ | ~~NA~~ | ~~NA~~ | ~~NA~~ |
| 63 | female | (-) | ~~NA~~ | ~~NA~~ | ~~NA~~ | ~~NA~~ | ~~NA~~ |
| 64 | male | (-) | ~~NA~~ | ~~NA~~ | ~~NA~~ | ~~NA~~ | ~~NA~~ |
| 64 | male | (-) | ~~NA~~ | ~~NA~~ | ~~NA~~ | ~~NA~~ | ~~NA~~ |
| 66 | male | (-) | ~~NA~~ | ~~NA~~ | ~~NA~~ | ~~NA~~ | ~~NA~~ |
| 66 | male | (-) | ~~NA~~ | ~~NA~~ | ~~NA~~ | ~~NA~~ | ~~NA~~ |
| 67 | male | (-) | ~~NA~~ | ~~NA~~ | ~~NA~~ | ~~NA~~ | ~~NA~~ |
| 61 | female | (-) | ~~NA~~ | ~~NA~~ | ~~NA~~ | ~~NA~~ | ~~NA~~ |
| 57 | female | (-) | ~~NA~~ | ~~NA~~ | ~~NA~~ | ~~NA~~ | ~~NA~~ |
| 49 | female | (+) | 250.4 | 0.1 | 2 | 1 | 3.3 |
| 38 | male | (+) | 133.4 | 0.8 | 0.2 | 1.1 | 5.3 |
| 39 | male | (+) | 375 | 1.45 | 0.3 | 0.92 | 5.05 |
| 40 | male | (+) | 982 | 2.33 | 0.1 | 0.14 | 4.13 |
| 49 | male | (+) | 105 | 2.6 | 1 | 0.28 | 4.23 |
| 63 | male | (+) | 453 | 0.4 | 0.1 | 0.24 | 1.88 |
| 64 | female | (+) | 234 | 0.1 | 0.13 | 1.1 | 3 |
| 68 | male | (+) | 109 | 0.3 | 1 | 1 | 3 |
| 59 | male | (+) | 741 | 0.11 | 1 | 1.2 | 6.4 |
| 58 | male | (+) | 1011 | 0.39 | 0.9 | 1 | 9 |
| 40 | male | (+) | 245 | 0.28 | 2.5 | 0.04 | 3 |
| 57 | male | (+) | 743 | 1.2 | 2.1 | 0.1 | 2.21 |
| 56 | female | (+) | 233 | 1.3 | 1 | 0.1 | 3 |
| 55 | female | (+) | 453 | 0.44 | 1 | 2.45 | 4.4 |
| 59 | male | (+) | 190 | 0.28 | 1 | 3.05 | 3 |
| 60 | male | (+) | 367.11 | 0.9 | 1 | 1.88 | 4.51 |
| 61 | male | (+) | 320 | 2.2 | 1 | 0.1 | 4.9 |
| 56 | male | (+) | 110.05 | 0.1 | 2.24 | 0.1 | 3 |
| 70 | male | (+) | 200 | 0.1 | 0.8 | 0.08 | 6.22 |
| 46 | female | (+) | 759 | 0.22 | 0.22 | 1.01 | 5.03 |
| 56 | female | (+) | 367 | 0.39 | 0.23 | 1 | 5.55 |
| 70 | female | (+) | 985 | 1.3 | 0.1 | 1 | 5.53 |
| 46 | male | (+) | 107 | 0.27 | 0.98 | 0.2 | 5.01 |
| 57 | male | (+) | 148.11 | 0.8 | 2.1 | 1 | 4 |
| 50 | male | (+) | 246.08 | 0.9 | 1.3 | 1 | 4.9 |
| 60 | male | (+) | 312.04 | 0.8 | 1.2 | 1 | 3.29 |
| 60 | male | (+) | 633 | 0.2 | 0.32 | 0.1 | 6.35 |
| 60 | female | (+) | 302 | 0.1 | 1.1 | 0.1 | 4.93 |
| 61 | female | (+) | 333 | 1.45 | 1.39 | 0.45 | 2 |
| 62 | male | (+) | 486 | 1.89 | 1.92 | 2.33 | 2 |
| 55 | male | (+) | 488 | 1.09 | 1 | 1.21 | 4 |
| 55 | male | (+) | 390 | 0.33 | 0.44 | 0.6 | 6 |
| 55 | male | (+) | 400.71 | 2.78 | 0.52 | 0.1 | 7 |
| 61 | male | (+) | 820.09 | 2.01 | 1 | 0.1 | 8 |
| 45 | male | (+) | 800 | 1.99 | 1.34 | 0.75 | 4.9 |
| 50 | male | (+) | 105 | 0.05 | 1.34 | 1 | 6 |
| 50 | male | (+) | 244 | 0.29 | 1 | 0.1 | 4 |
| 58 | male | (+) | 140 | 0.34 | 1 | 0.1 | 4 |
| 55 | male | (+) | 221 | 0.09 | 2.1 | 1 | 6 |
| 55 | female | (+) | 233 | 0.1 | 2.5 | 1 | 4 |
| 61 | female | (+) | 783 | 0.1 | 1 | 0.2 | 4 |
| 67 | male | (+) | 600 | 0.4 | 2.4 | 0.2 | 5.7 |
| 66 | male | (+) | 284 | 2.5 | 2.2 | 0.2 | 5.8 |
| 68 | male | (+) | 100.1 | 1.6 | 1 | 1 | 5 |
| 72 | male | (+) | 100.46 | 1.4 | 1.04 | 0.8 | 4.9 |
| 70 | male | (+) | 204 | 1.3 | 1 | 1 | 6 |
| 68 | female | (+) | 222 | 1.2 | 2 | 0.2 | 3.3 |
| 54 | female | (+) | 399 | 1 | 2 | 0.2 | 4.05 |
| 71 | female | (+) | 102 | 1 | 1.1 | 1 | 7 |
| 45 | female | (+) | 484 | 1 | 3.65 | 0.2 | 3.88 |
